# Inter-subunit interactions drive divergent dynamics in mammalian and *Plasmodium* actin filaments

**DOI:** 10.1371/journal.pbio.2005345

**Published:** 2018-07-16

**Authors:** Ross G. Douglas, Prajwal Nandekar, Julia-Elisabeth Aktories, Hirdesh Kumar, Rebekka Weber, Julia M. Sattler, Mirko Singer, Simone Lepper, S. Kashif Sadiq, Rebecca C. Wade, Friedrich Frischknecht

**Affiliations:** 1 Integrative Parasitology, Center for Infectious Diseases, Heidelberg University Medical School, Heidelberg, Germany; 2 Molecular and Cellular Modeling, Heidelberg Institute for Theoretical Studies (HITS), Heidelberg, Germany; 3 Center for Molecular Biology (ZMBH), DKFZ-ZMBH Alliance, Heidelberg, Germany; 4 Interdisciplinary Center for Scientific Computing (IWR), Heidelberg, Germany; Beatson Institute for Cancer Research, United Kingdom of Great Britain and Northern Ireland

## Abstract

Cell motility is essential for protozoan and metazoan organisms and typically relies on the dynamic turnover of actin filaments. In metazoans, monomeric actin polymerises into usually long and stable filaments, while some protozoans form only short and highly dynamic actin filaments. These different dynamics are partly due to the different sets of actin regulatory proteins and partly due to the sequence of actin itself. Here we probe the interactions of actin subunits within divergent actin filaments using a comparative dynamic molecular model and explore their functions using *Plasmodium*, the protozoan causing malaria, and mouse melanoma derived B16-F1 cells as model systems. Parasite actin tagged to a fluorescent protein (FP) did not incorporate into mammalian actin filaments, and rabbit actin-FP did not incorporate into parasite actin filaments. However, exchanging the most divergent region of actin subdomain 3 allowed such reciprocal incorporation. The exchange of a single amino acid residue in subdomain 2 (N41H) of *Plasmodium* actin markedly improved incorporation into mammalian filaments. In the parasite, modification of most subunit–subunit interaction sites was lethal, whereas changes in actin subdomains 1 and 4 reduced efficient parasite motility and hence mosquito organ penetration. The strong penetration defects could be rescued by overexpression of the actin filament regulator coronin. Through these comparative approaches we identified an essential and common contributor, subdomain 3, which drives the differential dynamic behaviour of two highly divergent eukaryotic actins in motile cells.

## Author summary

Actin is one of the most abundant and conserved proteins across eukaryotes. Its ability to assemble from individual monomers into dynamic polymers is essential for many cellular functions, including division and motility. In most cells, actin is able to form long and stable filaments. However, an actin of the malaria-causing parasite *Plasmodium*, while having a very similar monomer structure to actins from other eukaryotes, forms only short and unstable filaments. These short and dynamic filaments are crucial in allowing the parasite to move very rapidly in tissue. Here we investigated the basis of these differences. We used molecular dynamics simulations of actin filaments to investigate the actin–actin interfaces in filaments from *Plasmodium* and rabbit. We next engineered parasites to express chimeric actins that contained different parts of rabbit and parasite actin and thereby identified actin residues important for parasite viability and progression across the life cycle. We could rescue the most prominent defect specifically with overexpression of the actin binding protein coronin. This suggests that the more stable actin harms the parasite and that coronin helps in recycling filaments. By screening the effects of actin chimeras in mammalian cells, we also identified regions that allow these different actins to efficiently interact with each other. Taken together, our results improve our understanding of the interactions required for actin to incorporate into filaments across divergent eukaryotes.

## Introduction

Actin is a highly conserved cytoskeletal protein with essential roles in cell division, contraction, and motility. Cell motility is an important process in biological development, cancer metastasis, and tissue penetration by both immune cells and pathogens. Many eukaryotic cell types have the ability to move continuously in a substrate-dependent ameboid manner in both 2D and 3D environments, typically by deforming their cellular shape and protruding the cell’s leading edge [1–3]. Gliding motility is an alternative mode of locomotion displayed by some bacteria and protists that is independent of cell shape changes [4–6]. The malaria-causing parasite *Plasmodium* employs gliding motility in several phases of its life cycle. Gliding is essential for successful penetration of host organs such as the mosquito midgut and salivary glands as well as the mammalian liver [7]. Prior to liver infection, high motility speeds of 1–3 μm/s allow the parasite to escape from the dermis, where it is deposited by the biting mosquito, and thereby evade infiltrating neutrophils, the fastest migrating human cells, which move much slower (1–5 μm/min) [8,9]. Despite these striking differences in locomotion modes and speeds, the fundamental requirement for these cells is the dynamic turnover of actin filaments [10–12].

**Fig 1:**
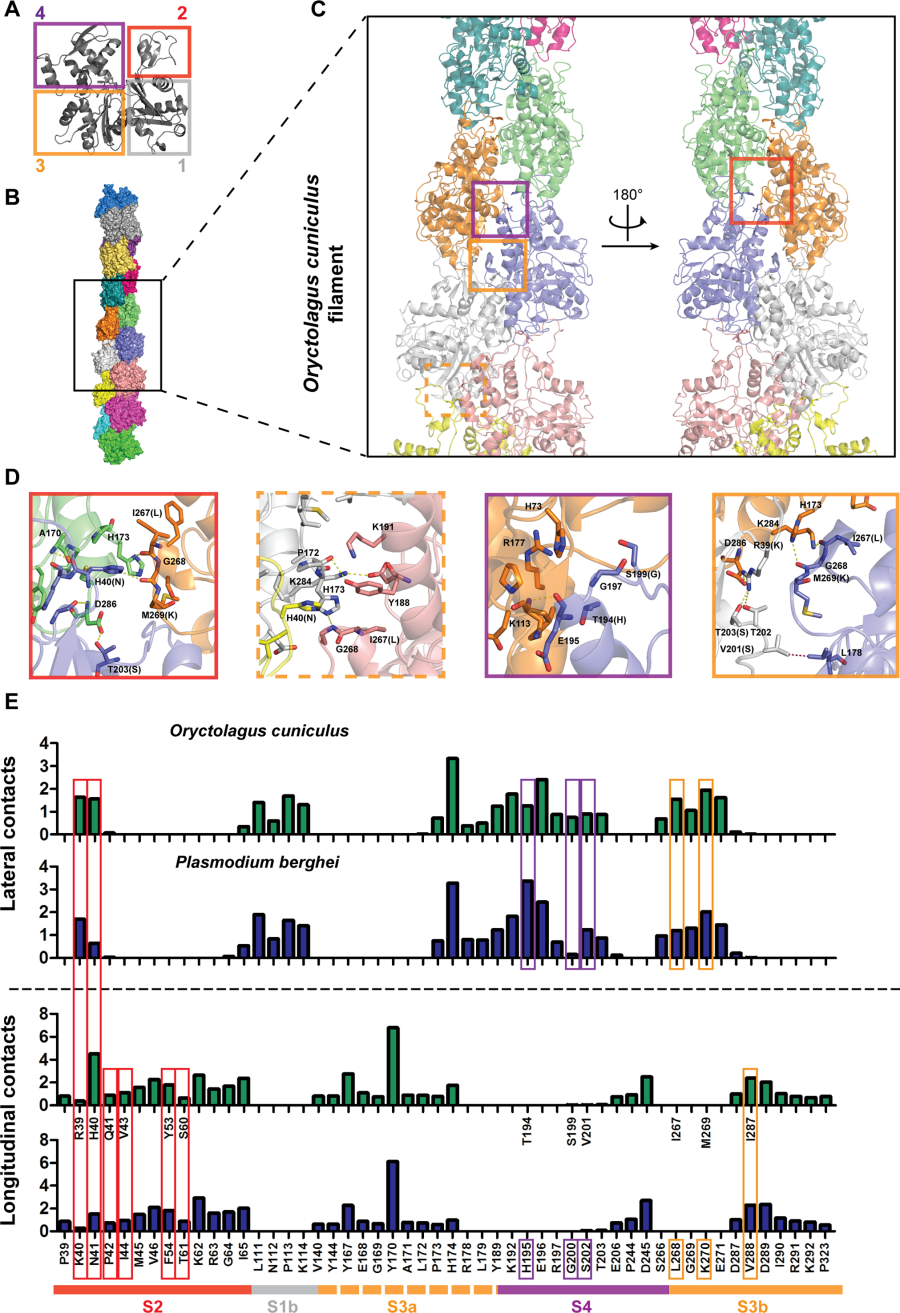
Sequence divergence in prominent lateral and longitudinal contact sites between actin subunits of different species. (A) Actin monomer with highlighted subdomains (numbers 1–4 boxed in different colours). (B) Snapshot from a simulation of a coarse-grained model of a rabbit alpha actin filament composed of 15 subunits. Different subunits are represented in different colours from subunit A (bottom) to subunit O (upper). (C) A close-up of the central part of the actin filament. Different colour boxes highlight regions of prominent inter-subunit contacts in the filament. Colours correspond to the different actin subdomains. (D) Respective colour boxes present close-up interactions of boxes in (C). (E) Bar graphs indicating the most prominent residues for each species and their mean number of contacts with lateral and longitudinal subunits. Divergent residues between species are indicated with coloured boxes of their respective subdomain locations, as in (A–C). Green bars are for rabbit alpha actin and blue bars indicate the contacts from *Plasmodium berghei*. Contacts are averaged across simulation time as described in Materials and methods. All unlabelled residues in the rabbit longitudinal graph indicate conservation between the two species. Lateral contact graphs have the same sequence content as longitudinal and are therefore not displayed. Underlying data can be found in S1 Data. Note that contact sites are similar between species. Nonetheless, differences in residues between species exist in these regions, as indicated by highlighting by the boxes coloured by interface region.

The actin monomer has a highly conserved structure that consists of four subdomains and a central nucleotide (adenosine triphosphate [ATP], adenosine diphosphate with inorganic phosphate [ADP + Pi], or adenosine diphosphate alone [ADP]) binding cleft (Fig 1A) [13]. Actin possesses the ability to self-assemble from monomers (G-actin) to form filaments (F-actin), which in turn can form higher order filamentous structures. Particular regions in the actin subdomains, such as the hydrophobic plug (H-plug) of subdomain 3 and the highly flexible DNAse I-binding loop (D-loop) of subdomain 2, as well as the nucleotide state, have been implicated as major contributors to the formation and stability of filaments [14–16]. Actin isoforms of most eukaryotes inherently form long and stable filament structures (greater than 1 μm in length), display high sequence conservation across species (>90% similarity from yeast to humans), and are regulated by a large set of actin binding proteins (ABPs) [17]. These ABPs can affect the monomer-filament ratio and fulfil a variety of regulatory roles, including but not limited to nucleation and elongation (e.g., formin), monomer binding (e.g., profilin), filament binding (e.g., coronin), and filament severing (e.g., cofilin/actin depolymerising factor [ADF] family). In contrast, protozoan parasites express divergent actins (60%–80% identity with vertebrate actins) that typically differ in their ability to form actin filaments. *Giardia*, trypanosomid, and apicomplexan actins are refractory to actin polymerisation modulating compounds, such as latruculin, and their structures are difficult to visualise with phalloidin [18–21]. Similarly, actin 1 of *Plasmodium* has fundamental shifts in its functional properties: despite a similar monomer tertiary structure [22] to mammalian actin, *Plasmodium* actin only forms short filaments of approximately 100 nm in length, has a noncanonical filament structure that is dynamically unstable [16,22–28], displays slow polymerisation yet rapid depolymerisation rates [29], and is regulated by a highly reduced set of predicted ABPs [30]. Such altered properties are crucial for intracellular parasite growth [31–33] and efficient parasite motility [12,31,33–35]. These fundamental differences make *Plasmodium* actin a useful model for the comparative assessment of key amino acid residues that result in altered filament properties. Here, we combined multiscale molecular dynamics (MD) simulations with three newly developed molecular genetic screens to identify the contribution of particular amino acid residues to the altered properties of canonical rabbit actin and the evolutionarily distant *Plasmodium* actin 1. The results reveal a distinct region in subdomain 3 as the primary contributor to divergent actin behaviour and filament incorporation.

## Results

### Discrete regions of the subunit–subunit interfaces in actin filaments are divergent between species

*Plasmodium* actin 1 is one of the most divergent eukaryotic actins known (80% sequence identity compared to rabbit alpha actin, S1 Fig). To assess the structural and dynamic differences between actin monomers from parasites, as represented by *Plasmodium* actin 1, and canonical mammalian actin monomers, as represented by rabbit actin, we first employed all-atom MD simulations. Apart from a more flexible region in subdomain 4, comparison of the breathing dynamics of the actin monomers revealed no marked differences in behaviour between the actins from the two species (S2 Fig). To assess actin filament dynamics, we constructed 15-mer *Plasmodium* and rabbit actin filaments based on electron microscopy (EM) data for the rabbit alpha actin filament [36] and performed five coarse-grained (CG) MD simulations of 10-μs duration for each filament. These simulations recapitulated the modified filament architecture observed in previous EM studies, in terms of alpha angle and subunits per crossover [16,22,27] (S3 Fig). Moreover, our models allowed for the comparative assessment of the dynamics of the molecular interactions both within and between subunits in the filaments. Comparison of the filament models for the two species revealed that both filaments displayed essential common interaction hot spots in regions involved in intermolecular contacts (Fig 1B–1E). Two interaction regions in subdomains 1 and 3 (designated S1b and S3a) are highly conserved across species (over 90% sequence identity of *Plasmodium* compared to rabbit alpha actin). Other interaction regions corresponded to sequences in subdomains 2, 3b, and 4 (Fig 1E, overall sequence identities of 78%, 72%, and 77%, respectively). Interestingly, within these regions there are residue differences between *Plasmodium* and rabbit actin filaments and thus altered contacts between filament subunits (Fig 1E). Thus, whereas the locations of the interfaces are conserved, the residues are, on average, more divergent at the interfaces than elsewhere (S1 Table).

### Specific regions of the parasite actin sequence affect host organ penetration and efficient motility

**Fig 2:**
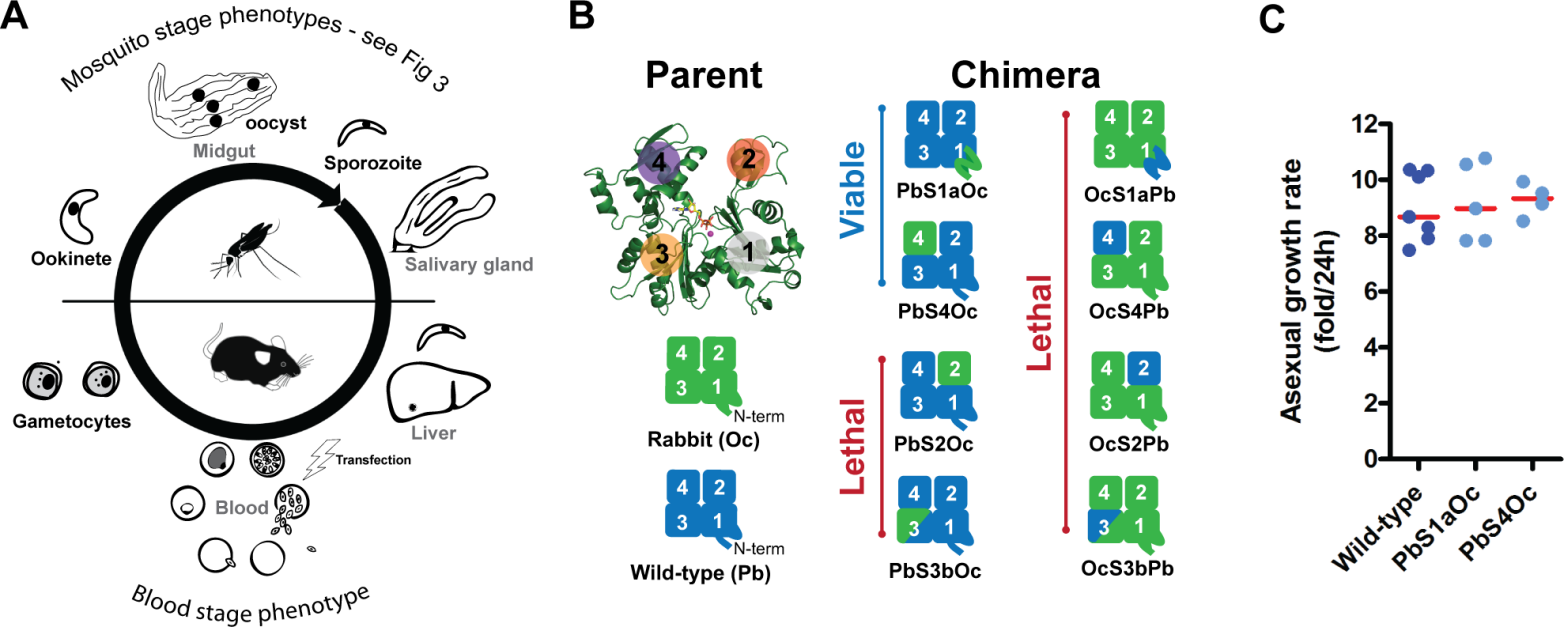
Unbiased allelic replacement screen of the *Plasmodium* endogenous actin 1 locus reveals that swaps of some divergent regions produce viable blood stage parasites. (A) A simplified schematic overview of the malaria parasite life cycle indicating various forms of the parasite (black) and the tissues penetrated (grey font). Note that genetic modifications by transfection are performed in the asexual blood stage forms. (B) Chimera design between rabbit and *Plasmodium* actins. Numbers indicate actin subdomains 1–4; colours on the structure correspond to those in Fig 1. Replacement of the endogenous actin with actin chimeras led to two viable parasite lines, while six chimeras are lethal. (C) A single blood stage parasite was injected intravenously (IV), parasitemia monitored, and daily asexual growth rates calculated. Parasite lines that were viable after transfection grew at similar rates when compared to the wild-type control. Red line indicates median. Underlying data can be found in S1 Data. Oc, *Oryctolagus cuniculus* (rabbit); Pb, *Plasmodium berghei*.

We sought to assess the contribution of these divergent regions to the altered behaviour of actin species in their cellular context. As a first unbiased screen, chimeras containing exchanges between *Plasmodium* and mammalian actin were generated and tested for their ability to replace the endogenous *actin 1* gene in the parasite (Fig 2A and 2B). To do so, we established a two-step genetic methodology that allowed for replacement of the endogenous copy while leaving the desired locus selection marker free and with minimal flanking changes in the parasite genome (S4 Fig). For rabbit actin-based chimeras, our screening approach revealed that any changes to this actin were insufficient to obtain viable parasite lines, strongly indicating that no one region is capable of full restoration of parasite-like actin function from a canonical backbone. Even for exchanges introduced into the parasite actin, the majority of the parasite actin chimeras were unable to functionally replace the endogenous gene. Importantly, changes to subdomains 2 and 3 of the parasite actin resulted in parasite death, indicating that these regions provide essential features required for parasite blood stage growth (the stage at which the genetic manipulations are performed, Fig 2A). Further, mutants with single point mutations in these subdomains (P42Q and K270M) were also lethal, strongly implicating the essentiality of these regions for normal parasite function in the blood stages of infection. However, two of the most divergent regions could be exchanged with the canonical mammalian actin equivalent regions: an N-terminal domain swap (PbS1aOc, residues 1–33, 67% sequence identity with rabbit alpha actin with one additional acidic residue on its N-terminus) and a subdomain 4 replacement (PbS4Oc, residues 182–263, 77% sequence identity with rabbit alpha actin). Interestingly, these transgenic parasites displayed asexual growth rates in the blood that were comparable to the wild-type replacement control (Fig 2C), suggesting that modification of these regions does not significantly affect intracellular growth and erythrocyte invasion.

**Fig 3:**
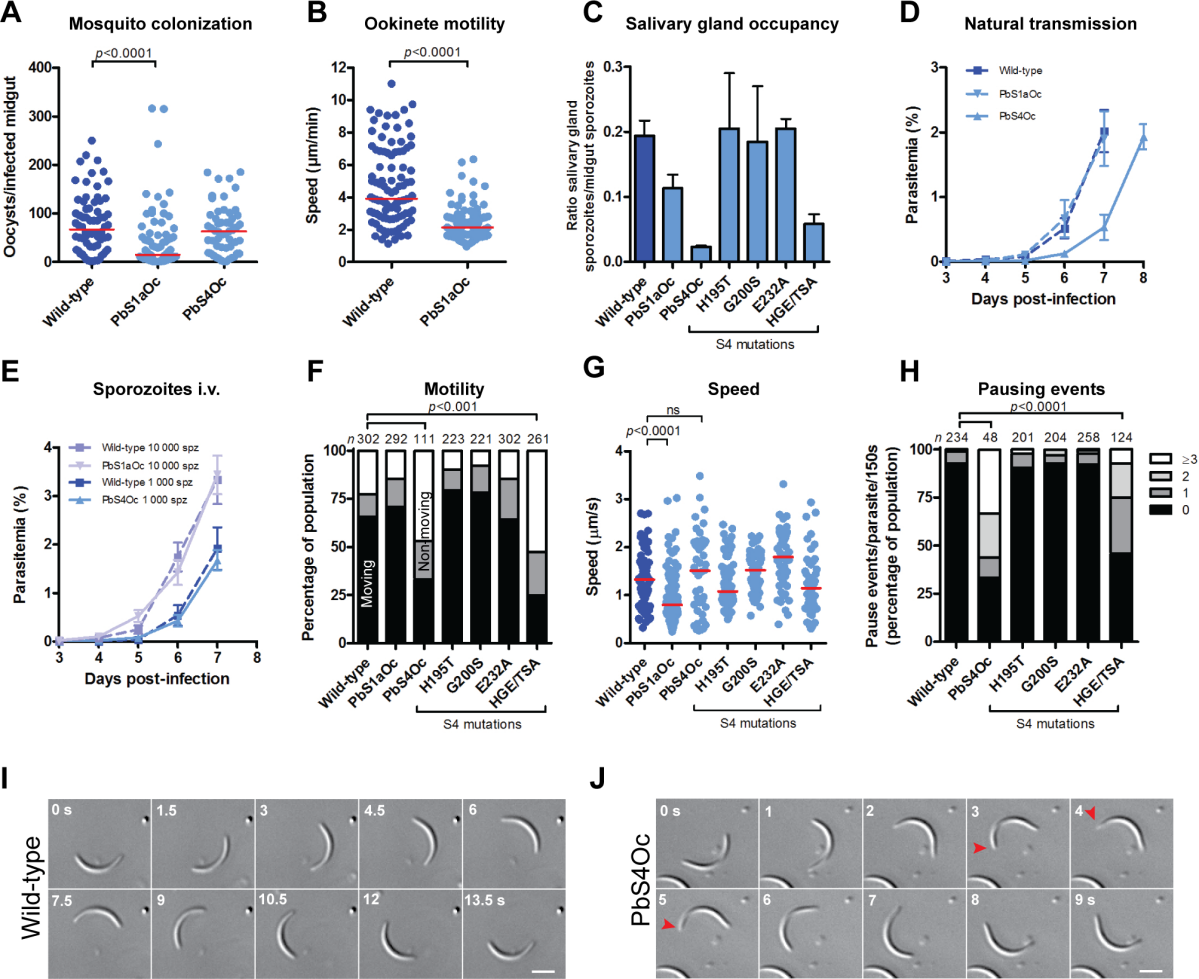
Exchange of actin subdomains 1 and 4 specifically affect mosquito stage organ penetration and motility. (A) PbS1aOc but not PbS4Oc showed a reduction in mosquito midgut colonisation compared to wild-type (Mann–Whitney test, *n* > 50 midguts, red line indicates median). (B) The reduced midgut colonisation of PbS1aOc can be attributed to reduced ookinete speed. PbS1aOc ookinetes (modified N-terminus) show approximately 50% reduction in median speed (red line) compared to wild-type controls (Mann–Whitney test). (C) Both PbS1aOc and PbS4Oc parasite lines as well as a triple mutant of subdomain 4 (HGE/TSA) displayed reduced salivary gland organ penetration. Data represented as mean ± standard error (*n* ≥ 2 independent mosquito feeds). (D) Transmission studies of wild-type and actin chimera harbouring parasites show that all lines are capable of reinfecting the mammalian host by mosquito bite. In natural transmission studies, there was a 1-d delay in emergence from the liver for PbS4Oc, which is most likely explained by the 10-fold reduction in parasite numbers residing in the glands (see panel c). Thus, fewer parasites would be deposited into the mammalian host dermis. (E) IV injection (1,000 or 10,000 sporozoites) resulted in an emergence of parasites from the liver at a comparable rate. Because of a reduced capacity of PbS4Oc to occupy glands, 1,000 sporozoites were injected IV and blood stage parasites emerged in a comparable manner to wild-type controls. In (D) and (E) data represented as mean ± standard error. (F) Reduction of motility in PbS4Oc and the triple mutant but not PbS1aOc salivary gland derived sporozoites. (Fisher’s exact test, *n* is the total number of parasites counted per group.) Black, grey, and white bars: efficiently, partially, and non-motile but attached sporozoites, respectively. (G) Reduction of PbS1aOc but not PbS4Oc sporozoite speed (Mann–Whitney test, red line indicates median). Individual data points reflect the average speed of individual parasites tracked over 150 s. (H) Aberrant ‘stop-go’ motility pattern of PbS4Oc and HGE/TSA sporozoites showing increased pausing during gliding (Fisher’s exact test, *n* is the total numbers of parasites counted per group). Underlying data can be found in S1 Data. (I) Time-lapse images showing normal motility in wild-type controls. Note the smooth counterclockwise movement with time. Numbers indicate time in seconds; scale bar: 5 μm. (J) Representative time-lapse images of gliding PbS4Oc sporozoites. Red arrowheads indicate a pause and reversal event. Numbers indicate time in seconds; scale bar: 5 μm.

The malaria parasite needs to propagate and disseminate in a variety of tissue environments and different temperatures, suggesting that the required actin dynamics of these other stages could be different [37,38]. We thus tested if the PbS1aOc and PbS4Oc chimeric lines were effective in infection of *Anopheles* mosquitoes. After transmission to the mosquito midgut, the parasite develops into midgut-penetrating ookinetes, which can traverse the epithelia to establish oocysts on the basal lamina (Fig 2A). These oocysts produce thousands of sporozoites that are subsequently released into circulation and actively invade the insect’s salivary glands [7]. PbS1aOc produced slower moving ookinetes and reduced oocyst numbers (Fig 3A and 3B). Furthermore, while the numbers of sporozoites in the mosquito circulation were within the usual range, suggesting normal oocyst development, a marked reduction in salivary gland occupancy and sporozoite motility was observed. PbS1aOc displayed a 50% reduction in parasite numbers in the salivary glands (Fig 3C). While this line was capable of infecting naive mice at similar rates to wild-type controls (Fig 3D and 3E) and showed a similar motile population compared to wild-type controls (Fig 3F), these parasites moved at a lower average speed (Fig 3G). Thus, a modification of the N-terminal region of actin, with a concomitant increase in the number of acidic residues, resulted in a decreased motility of two different parasite stages, which affected their ability to penetrate the organs of the mosquito.

Alteration of the amino acid residue composition of subdomain 4 (PbS4Oc) resulted in a much more pronounced defect in salivary gland invasion (Fig 3C). Again, this line was still able to cause infection in mice by natural mosquito transmission, although the decreased numbers of parasites in these glands resulted in a concomitant delay in infection (Fig 3D and 3E). The motile population of salivary gland–resident parasites was reduced and these moved in a discontinuous fashion (Fig 3F–3H). Interestingly, these parasites often paused during motility, a phenomenon not yet described for any *Plasmodium* mutant (Fig 3H). Intriguingly, such events were often accompanied by short reversals in direction and detachment at the rear of the parasite (Fig 3I and 3J, S1 Movie and S2 Movie). While pausing did not change the average speed compared to wild-type, there was an increased range of speeds obtained by individual parasites (Fig 3G).

Changing the subdomain 4 region involved the alteration of 20 amino acid residues to canonical equivalents. Strikingly, the pausing phenotype was also observed by combined modification of only three residues in this region (three combined parasite to mammalian residue mutants H195T, G200S, and E232A: mutant HGE/TSA) (Fig 3 and S5 Fig). CG MD simulations with this triple mutant indicate that a change of these three residues results in a shift of filament parameters toward rabbit actin (S3 Fig) and an atypical interaction profile that is not observed in either species: residues F54 and Y189 display enhanced contacts with Y170 and H174, respectively, which could have consequences for actin filament turnover (S6 Fig) [16,36]. Taken together, altering actin dynamics by mutation of nonessential actin regions results in parasites that, while behaving normally in the mammalian host, have prominent inabilities to effectively colonise the mosquito vector.

### Overexpression of F-actin binding protein coronin restores parasite mosquito colonisation in a mutated actin line

**Fig 4:**
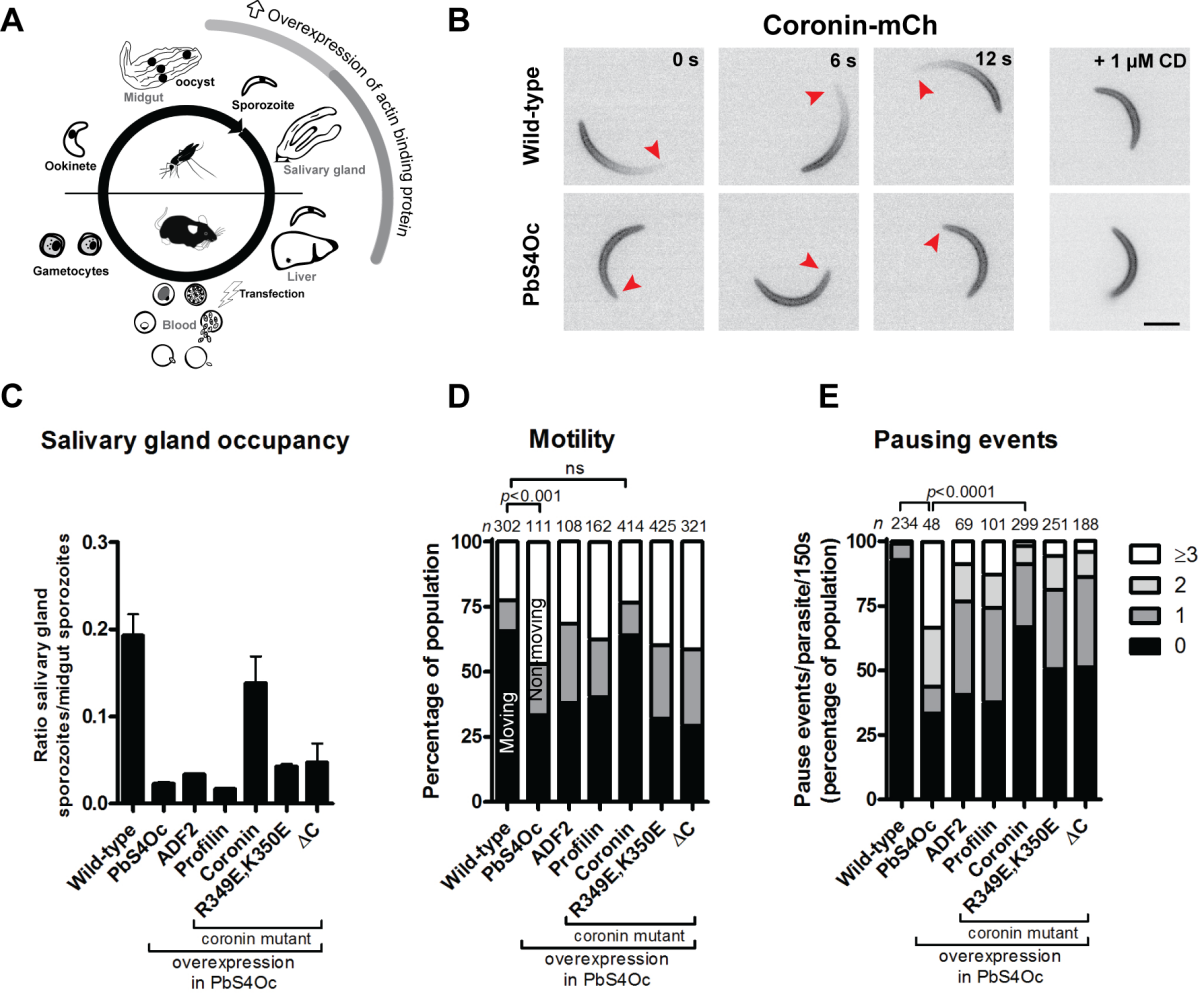
Coronin displays reduced binding, yet overexpression rescues the PbS4Oc phenotype. (A) Overexpression of actin binding proteins specifically in the sporozoite stage of the life cycle. (B) Coronin localises at the back of motile wild-type but along the cell periphery in PbS4Oc sporozoites or sporozoites treated with CD, suggesting reduced efficiency of coronin binding. Red arrowheads indicate the front of the sporozoites, scale bar: 5 μm. (C–E) Profilin and ADF2 overexpression were unable to restore organ penetration (c), motility (d), and reduce pausing (e), while coronin overexpression was able to partially rescue the phenotype observed in the PbS4Oc line. In (C) data represented by mean ± standard error. In (D) black, grey, and white bars: efficiently, partially, and non-motile but attached sporozoites, respectively. Fisher’s exact test comparing moving groups in [D] and 0 pause groups in [E]; *n* is the total numbers of parasites counted per group. A coronin actin binding mutant (K349E,R350E) and a coronin construct lacking the C-terminal region (yet having the intact actin binding domain, ΔC) were unable to rescue the phenotype, suggesting an interplay between N- and C-terminal regions to mediate rescue. Please note that the wild-type and PbS4Oc data are the same as from Fig 2C, 2F and 2H. Underlying data can be found in S1 Data. ADF2, actin depolymerising factor 2; CD, cytochalasin D; ΔC, coronin construct lacking C-terminal region; K349E,R350E, full length coronin actin binding mutant; mCh, mCherry.

*Plasmodium* actin cannot be visualised using typical imaging approaches [39], yet expressing the F-actin binding *Plasmodium* coronin fused with mCherry enabled visualisation of F-actin in motile sporozoites [37]. Coronin localises at the periphery of nonmotile sporozoites and relocalises to the rear in motile sporozoites in an actin filament–dependent fashion [37]. To investigate a possible change in localisation, we transfected PbS4Oc-expressing parasites with mCherry-tagged coronin under the control of a sporozoite-specific promoter (Fig 4A, S7 Fig). Curiously, coronin localised to the periphery in an actin-independent manner in these sporozoites resembling the localisation in nonmotile wild-type sporozoites or after treatment with the actin filament modulator cytochalasin D (Fig 4B). This indicates that the majority of the tagged coronin was unable to bind the modified actin, similar to observations with a coronin actin binding mutant and when parasites are treated with filament stabiliser, jasplakinolide [37]. Intriguingly, overexpression of coronin, but not overexpression of profilin and ADF2, rescued efficient motility and salivary gland penetration of PbS4Oc sporozoites (Fig 4C–4E). Overexpression of coronin in WT sporozoites also increased their capacity to move [37], suggesting that in PbS4Oc sporozoites, coronin overexpression is compensating for its reduced binding ability to a mutated actin. To further probe the domain contributions to this rescue, we tested whether mutations to coronin would improve motility and invasion of the PbS4Oc parasite line. Mutations in the N-terminal actin binding domain (coronin mutant R349E, K350E), as expected, could not rescue the PbS4Oc phenotype. We also tested if the actin binding N-terminal domain of coronin (residues 1–388, lacking the unique region and coiled-coil domain), which was shown to bind and bundle actin [40], affected motility and invasion. This also could not improve the invasion and motility of the PbS4Oc parasite, suggesting a cooperative involvement of both N- and C-terminal regions of coronin in mediating rescue (Fig 4C–4E).

### Exchanging subdomain 3 allows for improved incorporation into parasite filaments

**Fig 5:**
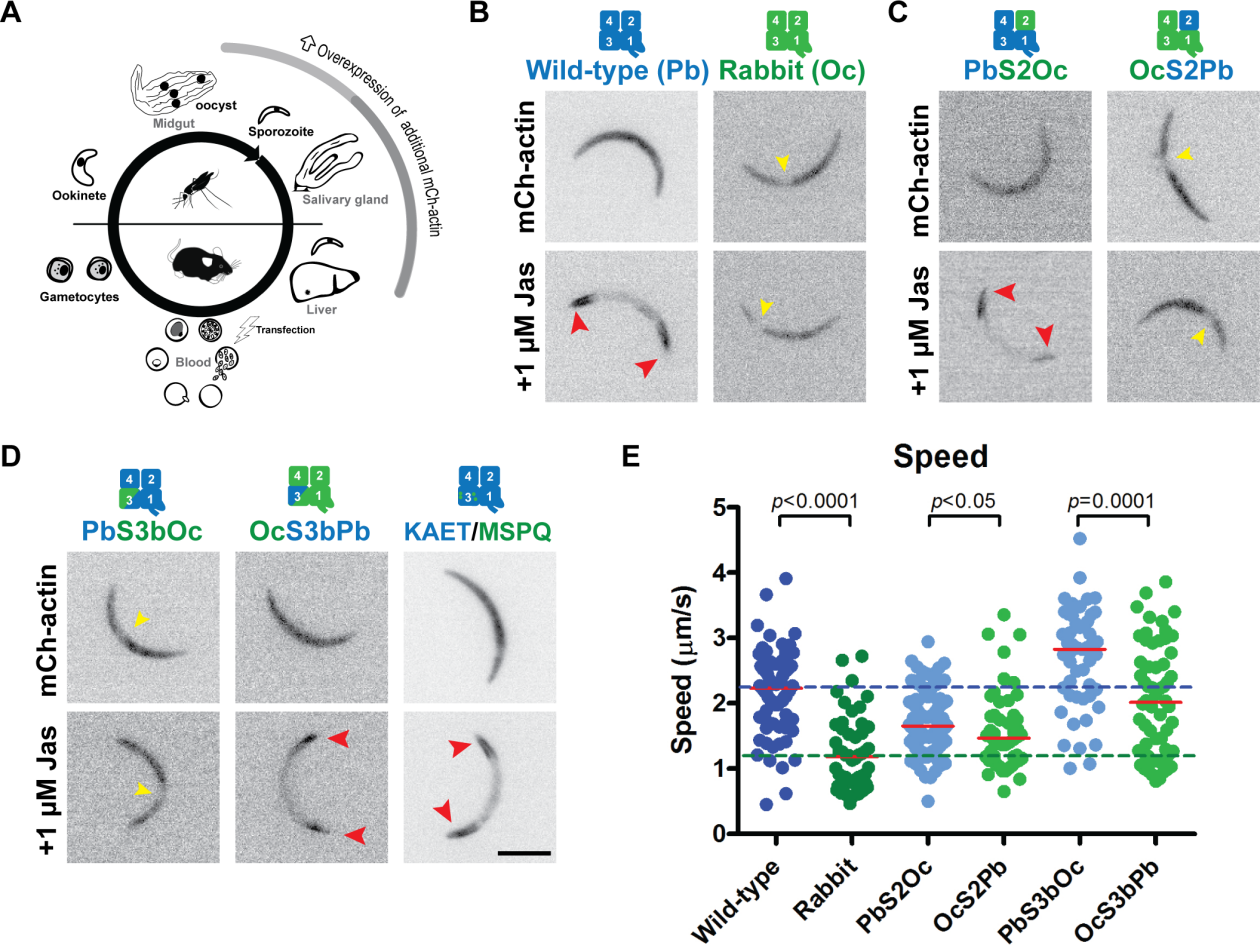
Actin chimera localisation in sporozoites reveals subdomain 3b as a key contributor for efficient filament incorporation. (A) Overexpression of ‘lethal’ actin chimeras specifically in the sporozoite stage of the life cycle. (B) Wild-type mCherry-actin is located throughout the cell and collects at the front and rear of the parasite upon Jas treatment (red arrowheads). In contrast, rabbit actin is absent from the nucleus (yellow arrowheads) and does not relocalise under Jas, suggesting reduced incorporation into *Plasmodium* filaments. (C) Expressing subdomain 2 chimeras in sporozoites resembles the localisation of their corresponding backbone controls. (D) Expressing subdomain 3 chimeras in sporozoites resulted in a ‘reciprocal’ localisation effect: rabbit actin backbone with a parasite actin subdomain 3 (OcS3bPb) gave parasite actin-like localisations and, inversely, parasite actin backbone with a rabbit actin subdomain 3 (PbS3bOc) gave a rabbit actin-like localisation pattern. This change in localisation was not solely dependent on amino acid changes in the H-loop and the most divergent residues elsewhere in the region because a protein containing these changes between species (KAET/MSPQ) resembled the wild-type parasite equivalent. Scale bar: 5 μm. (E) Sporozoites expressing rabbit actin or chimeras with a rabbit actin backbone moved significantly slower than sporozoites expressing parasite actin or chimeras with a parasite backbone, respectively. Individual data points reflect the average speed of individual parasites tracked over 150 s. Red lines indicate median. Dotted lines indicate the median values of wild-type and rabbit controls. Mann–Whitney test. Underlying data can be found in S1 Data. Jas, jasplakinolide; KAET/MSPQ, combined mutant K270M,A272S,E308P,T315Q.

Above, we identified subdomains 2 and 3 as key contributors to essential processes in blood stage parasites (Fig 2B). In order to assess the contribution of these regions to altered actin dynamics, we performed a second screen by expressing these variants (PbS2Oc, OcS2Pb [residues 34–71], PbS3bOc, and OcS3bPb [residues 264–338]) as mCherry-tagged additional copies under the control of a sporozoite stage-specific promoter (Fig 5A, S8 Fig and S9 Fig). This allowed for careful phenotypic characterisation in the parasite without the lethality observed in the initial screen. Tagged actins revealed interesting differences: an additional copy of parasite actin was present throughout the parasite cell, as expected [41]. Yet, mammalian actin was only cytoplasmic with a notable absence in the nuclear region (Fig 5B). Furthermore, the two ‘control’ parasite lines (expressing either parasite or canonical actin) differed in their response to jasplakinolide: parasite mCherry-actin accumulated at both the front and rear of the cell (Fig 5B), sites implicated for F-actin formation (in the case of formin 1, which is located at the front; S8 Fig) and turnover (rear) [37,40,41,42]. In contrast, mammalian mCherry-actin displayed no change in localisation (Fig 5B). This suggests that tagged mammalian actin is not readily incorporated into jasplakinolide-stabilised parasite F-actin in the sporozoite. We next assessed localisation and jasplakinolide responsiveness with the actin chimeras. Unexpectedly [22], the chimeras containing exchanges in subdomain 2 (a highly flexible and divergent region between species) were similar in behaviour to their corresponding controls (Fig 5C). In contrast, exchanging subdomain 3 resulted in reciprocal effects: a mammalian actin containing this corresponding parasite region (residues 264–338 with 21 changes; 72% sequence identity) resulted in a changed localisation under jasplakinolide, thus suggesting more efficient incorporation to endogenous filaments (Fig 5D). Changing the same region in the parasite actin resulted in a cellular distribution and jasplakinolide response similar to mammalian actin, suggesting that subdomain 3 contains the contacts necessary and sufficient for incorporation into divergent filaments. Importantly, the effect appears to be independent of the H-plug, as changing the two divergent amino acid residues on the loop and the two most divergent residues outside this loop back to residues in the canonical actin (K270M, A272S, E308P, and T315Q) still rendered an additional copy of actin that behaved similarly to *Plasmodium* actin (Fig 5D) [16]. Parasites expressing rabbit actin displayed a 50% reduction in parasite average speed (Fig 5E). Interestingly, all chimeras of *Plasmodium* actin consistently moved with a slightly higher average speed compared to their corresponding mammalian chimeras. OcS3bPb, the only mammalian actin chimera that responded to jasplakinolide treatment, moved at a similar speed to the wild-type *Plasmodium* actin control (Fig 5E).

### Actin subdomains 2 and 3 contribute to efficient parasite monomer incorporation in highly divergent cell systems

**Fig 6:**
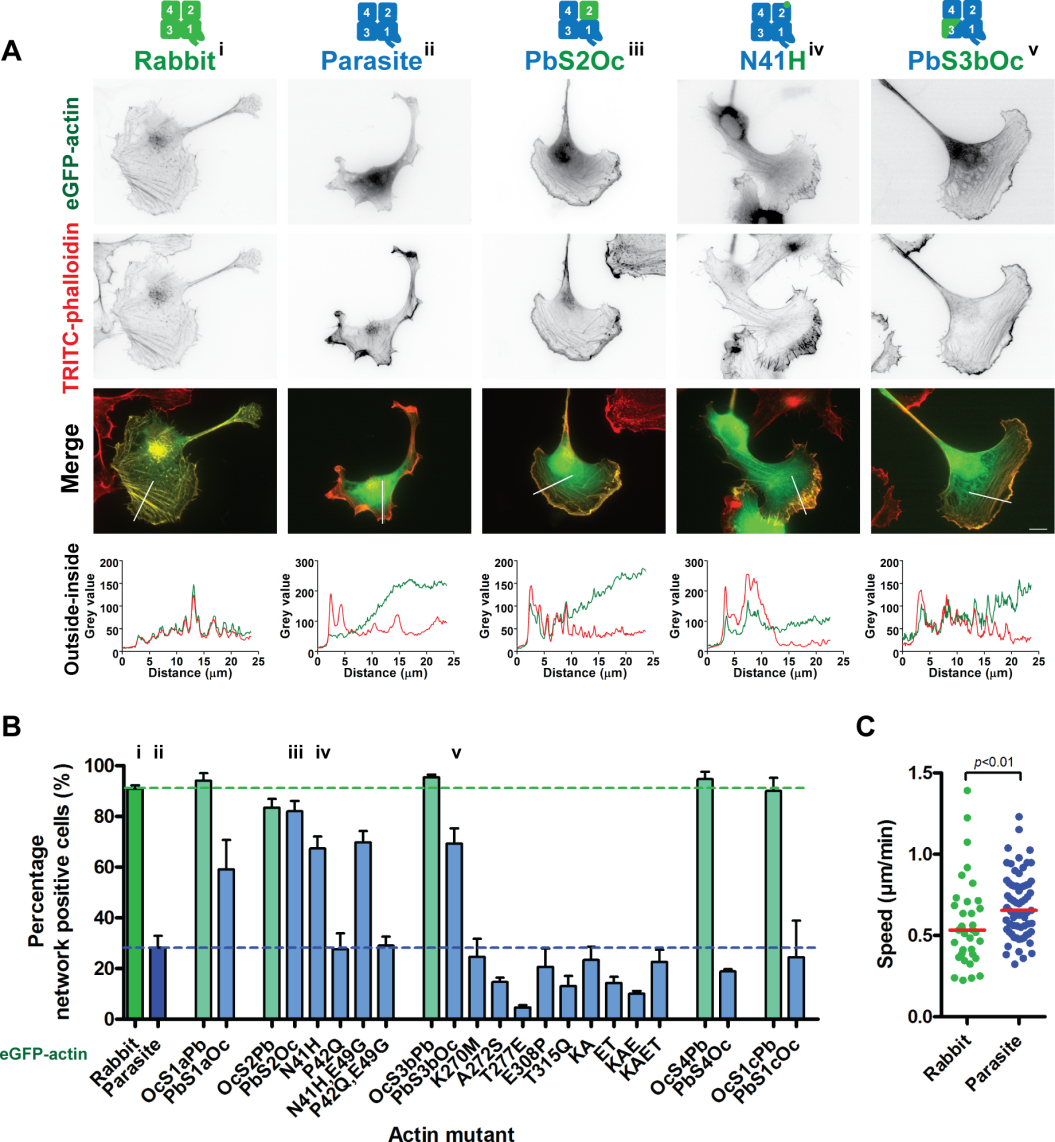
Actin chimera localisation in mammalian cells reveals residue 41 (N41H) and subdomain 3b as minimal contributors to efficient filament incorporation. (A) Representative images of B16-F1 cells transfected with different actins or actin chimeras. Mammalian eGFP-actin incorporates readily into the diverse filamentous structures of the cell, as revealed by phalloidin staining, while parasite eGFP-actin is not efficiently incorporated. Modification of either subdomain 2 or 3 leads to parasite actin being efficiently incorporated. A change of a single amino acid to its corresponding mammalian form (N41H) is sufficient for increased filament incorporation. Outside-inside line plots (along white bars) indicate eGFP signal overlap with filaments, particularly in the lamellipodial regions. Scale bar: 10 μm. (B) Quantification of filament incorporation as measured by percentage of cells positive for a GFP-positive actin network. Values given as mean ± standard error of the mean of at least three independent experiments. Roman numerals indicate the quantitation of the corresponding transfectants shown in (A). (C) B16-F1 cells expressing parasite actin move significantly faster than those expressing rabbit actin. Individual data points reflect the average speed of individual cells tracked. Red line indicates the median, Mann–Whitney test. Underlying data can be found in S1 Data. eGFP, enhanced green fluorescent protein; KA, K270M,A272S double mutant; ET, E308P,T315Q double mutant; KAE, K270M,A272S,E308P triple mutant; KAET, combined mutant K270M,A272S,E308P,T315Q; TRITC, tetramethylrhodamine-isothiocyanate.

To test whether the specific regions that we identified above are common contributors to actin dynamics in other cell types, we performed a third screen by transfecting the panel of chimeras into different mammalian cells as additional copies. Higher eukaryotic cells did not readily incorporate parasite actin into stable filamentous structures (Fig 6A and 6B, S10 Fig). Furthermore, cells containing parasite actin moved slightly faster in a random migration assay (Fig 6C). These observations indicate that malaria parasite actin can be used as a template to identify the minimum determinants required for filament incorporation in higher eukaryotic cells. Strikingly, changing subdomain 2 or 3 into the corresponding mammalian equivalent resulted in the parasite GFP-actin more readily incorporating into filament networks (Fig 6A and 6B). In contrast, exchanging individual regions of mammalian actin did not result in decreased incorporation, suggesting that more than one region can provide the required minimal contacts necessary for incorporation.

To identify key amino acid residues contributing to filament incorporation, multiple mutations (whereby parasite actin residues were converted to conserved mammalian counterparts) in both subdomain 2 and subdomain 3 were generated and analysed (Fig 6A and 6B). Consistent with the results of the parasite lines, single and multiple mutations of both the H-plug and regions typically on the surface of the actin filament in subdomain 3 did not result in improved filament incorporation, suggesting an extensive interface in the subdomain 3 region required for incorporation. Remarkably, a single residue change (N41H) in subdomain 2 of *Plasmodium* actin was sufficient to rescue filament incorporation (Fig 6A and 6B). This indicates that the presence of an imidazole group alone provides sufficient interaction for more efficient filament incorporation (Fig 6A and 6B). Thus, crucial differences between actin species, including the ability for a monomer to incorporate into divergent filaments, are due to the changes in particular divergent regions in specific subdomains only. Furthermore, subdomain 3 is the key common contributor to filament incorporation in two very diverse cellular systems.

## Discussion

### Differential functional requirements of *Plasmodium* and mammalian actin

While, structurally, the *Plasmodium* actin 1 monomer is similar to that of canonical mammalian actins [22], *Plasmodium* actin filaments are shorter and more dynamic [22,26,27]. Here, we have shown that *Plasmodium* actin is unable to efficiently incorporate into actin filaments of mammalian cells and that tagged mammalian and *Plasmodium* actin localise differently in parasites. Using MD simulations as well as chimeric and mutagenesis genetic screens, we identified the fundamental contributors that underlie these different characteristics. Our data independently confirm that these two actins occupy a similar structural space and identify divergent amino acid residues responsible for the differences in filament dynamics. *Plasmodium* was unable to tolerate allelic changes in subdomains 2 and 3, suggesting that these regions have crucial features required for parasite biology and are, particularly P42 and the H-plug, key contributors to contact sites. The data suggest that modifications of these two regions result in filaments that are too stable and thus lethal for the parasite, similar to the effects observed with jasplakinolide treatment or genetic ablation of the actin regulator, ADF1, and the alpha capping protein [31,32,43,44].

### Strong selection pressure on parasite actin in the mosquito

Unlike exchanges in subdomains 2 and 3, the parasite readily tolerated the exchange of highly divergent regions of subdomain 4 and the N-terminal residues (Fig 2). These parasite lines grew like wild-type parasites in the blood stage and were only affected in their progression in the mosquito host (Fig 3). Importantly, this indicates that red blood cell invasion, while dependent on actin [33], is more tolerant of minor changes to its sequence. This observation is consistent with ablating ABP expression having little effect on the parasite in the mammalian host but having a strong effect once the parasite infects the insect vector [37,44–48]. While other studies looked indirectly with actin regulators, our study investigated the core of the machinery itself across the life cycle, revealing that modest changes to dynamics have important consequences in mosquito colonisation. This indicates that the greatest selection pressure on the parasite actin sequence could be during active organ penetration of the mosquito.

Changes to the N-terminal sequence resulted in a consistent decrease in speed between the two parasite stages that employ gliding motility in the mosquito (Fig 3B and 3G). Given the well-known interaction between the actin N-terminus and myosin [49–56], it is reasonable to suggest that the decrease in cell speed is due to a reduced interaction between these two proteins. We thus propose that parasites containing this change in acidic residue content have reduced force transmission of actomyosin, which results in reduced average speeds. This hypothesis could be probed in vitro using classical myosin sliding filament assays with the respective *Plasmodium* machinery components [57].

Previous studies in yeast have indicated a relatively low threshold in opisthokonts to accommodate changes to its actin subdomain 1 sequence, in which the addition of a single acidic residue to the N-terminus rendered viable yet sick yeast cells, while two additional acidic residues were lethal for the cell [58]. In comparison, the addition of another acidic residue to parasite actin was surprisingly well tolerated (Figs 2 and 3): these parasites could complete the life cycle and move sufficiently well to cause infection (albeit at a lower overall efficiency in the mosquito). Interestingly, yeast cells with changes to subdomain 1 and the N-terminus were sensitive to changes in growth temperature [58–61]. Given the marked differences in body temperatures between mosquito and mammal, it is possible that changes in parasite actin sequence affect the well-tuned actin dynamics required at different temperatures. In vitro polymerisation assays with these mutants at both 37°C and 21°C would provide important insights into differences in the polymerisation kinetics at different temperatures. These detailed kinetic studies are especially interesting given that hybrid actins between rabbit muscle and yeast equivalents can produce unexpected changes to certain biochemical properties [58].

### Rapid filament recycling and continuous parasite movement

Interestingly, modifications of three amino acid residues in subdomain 4 rendered a parasite that frequently paused and reversed direction during migration (Fig 3H–3J). We propose that this change in motility is due to more stable actin filaments, which might be misoriented and hence allow partial rearward motility. Similar movements can be observed if *Toxoplasma* parasites are treated with high concentrations of jasplakinolide, a filament-stabilising drug [62]. We showed previously that coronin can bind to actin filaments in motile sporozoites but not to those treated with jasplakinolide [37]. Intriguingly, in the PbS4Oc sporozoites, coronin did not bind actin filaments and hence this parasite to some extent phenocopies jasplakinolide treatment. These parasites could also not enter salivary glands efficiently, for which well- tuned actin dynamics are important [37,45]. Yet, overexpression of coronin rescued both motility and invasion phenotypes (Fig 4). This indicates a central role for coronin in rapid filament recycling in the highly motile parasite. We propose that the discontinuous movements in subdomain 4 mutants (PbS4Oc and HGE/TSA) result from reduced actin filament recycling due to altered architecture and interactions at the filament interface, particularly by enhanced interactions of F54 and Y189. By alteration of subdomain 4 residues, the resulting contacts by conserved amino acid residues render the parasite actin filament less prone to disassembly as choreographed by coronin (S11 Fig).

Reduced invasion and aberrant motility were only rescued by full-length coronin, while mutated or truncated coronins were insufficient to rescue these phenotypes. This may suggest a cooperative requirement of the coronin domains for actin turnover (Fig 4C–4E). This result was somewhat surprising, because a recent publication on the biochemical properties of *P*. *falciparum* coronin suggested that much of the classical coronin function could be carried out by the N-terminal actin binding domain alone [40]. Our work indicates that, in the context of the *P*. *berghei* actin mutated sporozoite, both regions of coronin are required for full functionality. The mechanism by which coronin could be mediating *Plasmodium* filament recycling could be similar to that in higher eukaryotes. In these, coronin mediates a spatial-temporal recruitment of other filament regulators [63–65]. Coronin binds first and, depending on the nucleotide state of the actin [63,66], can serve to shield or allow access of ADFs to the filament. Binding of these additional factors together results in destabilisation of the filament. Given the close proximity of coronin and ADF on canonical filaments, it was also suggested that coronin could interact directly with ADF in the filament [64–67]. It is tempting to speculate that the rescue mediated by coronin could be due to a stepwise recruitment by coronin, with depolymerisation factors on the highly dynamic *Plasmodium* actin filament, which thus enhances severing. Our rescue experiments thus hint at a possibly conserved spatial-temporal coronin mechanism for filament regulation beyond what has currently been demonstrated for opisthokonts.

The C-terminal region of coronin appears important for this rescue, which suggests that this domain could allow for an interaction with other ABPs [68]. Indeed, it has been suggested for coronin of the related parasite *T*. *gondii* that the C-terminal coiled-coil domain could function as a molecular recruitment hub [69]. Alternatively, the C-terminal region simply provides an improved interaction with the filament, resulting in efficient turnover in the parasite. Together, our observations suggest interplay between both halves of *Plasmodium* coronin to mediate efficient rescue in motile sporozoites.

### In vivo screens reveal actin dynamics dependent on typically non-interfacial residues

**Fig 7:**
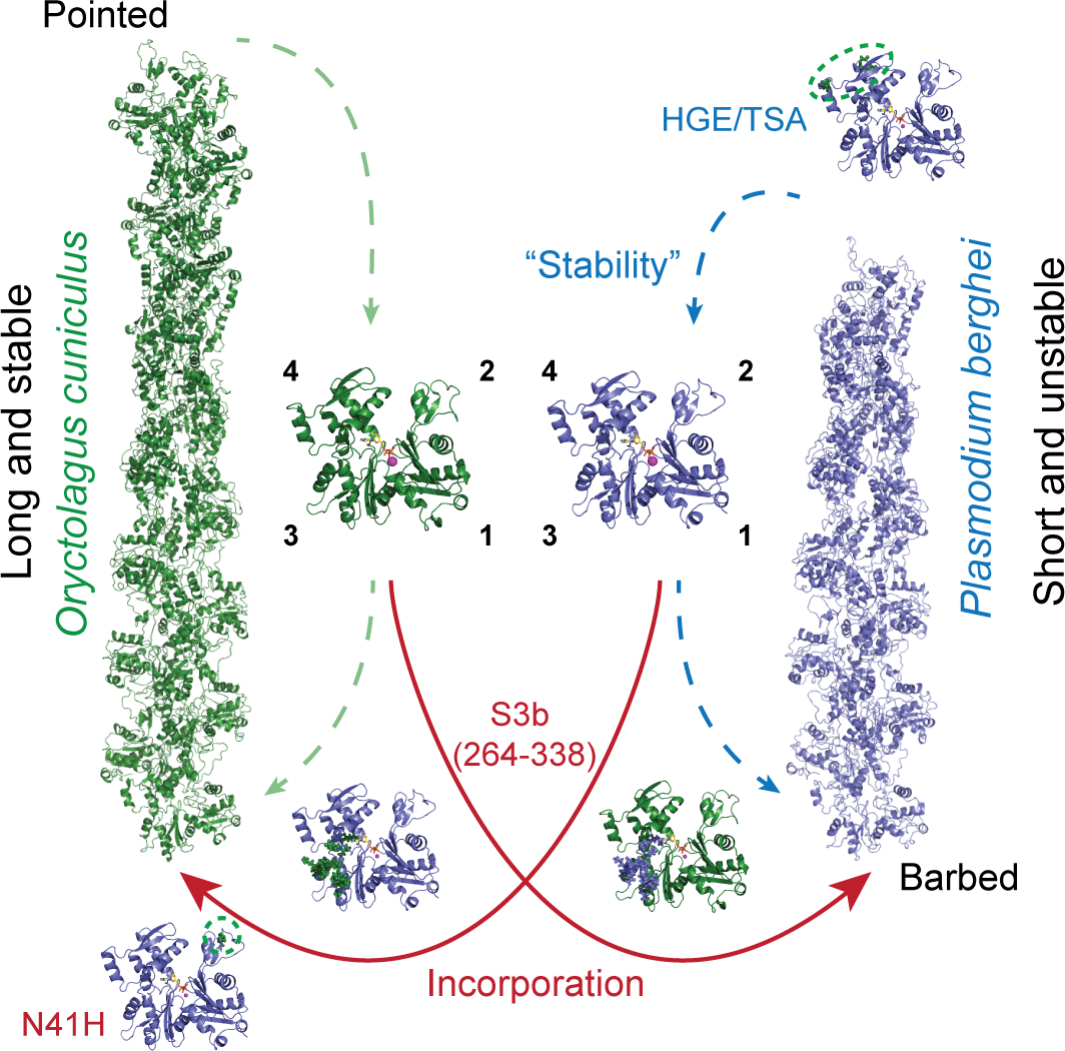
Specific regions of actin are primarily responsible for the differences between actins from *Plasmodium* and mammals. Residues or regions with blue labels correspond to contributors to filament stability, while red labels indicate contributors to incorporation of monomers into divergent filaments. In both cases, subdomain 3 (residues 264–338) is a critical contributor and allowed for divergent monomers to be reciprocally incorporated. Importantly, a change of a single amino acid residue (N41H) allowed for improved incorporation of *Plasmodium* actin into mammalian filaments. Dotted lines indicate the wild-type filament assembly and disassembly. For simplicity, we have only represented barbed end addition and pointed end dissociation. The lethal phenotypes of the S2 and S3b chimeras suggest that these regions also contribute to filament stability, similarly to the HGE/TSA mutant.

A recent high-resolution cryo-EM structure of the jasplakinolide-stabilised *Plasmodium* actin filament [16] is in good agreement with our models and generally fits with our biological observations. However, some of our in vivo observations could not be directly inferred from the static structure. For example, the combined mutation of H195 and G200 (to Thr and Ser, respectively) at a lateral interface in *Plasmodium* actin had no significant effect on the parasite across the entire complex life cycle. A third residue, E232, which is not at an interface, with the exception of one published structure [70], needed to be mutated in order for an effect on parasite biology to be observed, suggesting a dynamic interplay across several residues in this region (Fig 3C–3H). It is possible that E232 could provide a compensatory interaction when H195 and G200 are mutated. Our systematic approach, which spans from molecular models to cells, has thus identified novel cooperative interactions within actin filaments (Fig 7).

### Parasite actin provides molecular insights into monomer-to-filament incorporation

In this study, we made use of additional copy expression systems in highly divergent cells. It is important to note that these assays can only provide insights into monomer incorporation into filaments. Thus, we can make no direct inferences regarding whether each actin mutant, if present as the primary untagged actin in the cell, can fully complement wild-type function. For example, PbS2Oc was lethal as a replacement yet behaved similarly to wild-type *Plasmodium* actin as an additional copy in the parasite (Fig 5). Newly available genetic tools, such as inducible knockout systems [33], might be adapted to probe these otherwise lethal mutants for their functional consequences in cells.

Our observation that parasite actin does not efficiently incorporate into the mammalian cell actin network provided a useful tool in understanding the minimal requirements for network incorporation. Interestingly, subdomain 4 conversion of parasite actin did not improve network incorporation efficiency, while changing subdomain 2 or 3 resulted in a considerable incorporation into filamentous structures. Subdomain 2, with its highly dynamic extendibility, has been implicated to act as the primary interaction arm, after initial contact by subdomain 4, to allow the incoming monomer to bind to the barbed end [36]. The N41H mutant in *Plasmodium* actin subdomain 2 alone shows improved incorporation into mammalian actin filaments. Histidine has the capacity to have more contacts than asparagine and thus this position might be the key prominent interaction to allow optimal contact with the filament [16] (Fig 1E). Indeed, oxidation of H40 results in an actin monomer that is unable to polymerise [71,72]. Here, we extend this understanding to include this contact as important for an incoming monomer to dock onto a filament. We, with others, have shown that subdomain 3 provides important contact sites across the strand once the monomer has been incorporated and also for the incoming monomer to be included in the filament [15,36,73] (Fig 1 and Fig 7). Furthermore, we cannot exclude that subdomain 3 could be providing the required contact interface to allow for increased ABP-mediated incorporation [74]. We propose that having at least one of these important subdomain sites (2 or 3b) is sufficient for effective incorporation in higher eukaryotic cells. Identification of altered ABP binding between actin species could shed light on the primary factors responsible for monomer incorporation. Notably, there is a consistent feature between divergent systems: alteration of subdomain 3 allowed for a mammalian equivalent to be more efficiently incorporated into the highly divergent parasite cytoskeleton, indicating that this region could be a common ‘site of recognition’ across differently behaving actin species. This could be tested with other highly divergent actins to assess whether this region acts as a common recognition point across species.

### Conclusion

Through our comparative approaches, we have identified essential contributors to the differential behaviour of two highly divergent actin species (Fig 7). We have identified a region of subdomain 3 outside the H-plug as providing important contacts to allow an otherwise divergent actin to be more efficiently incorporated into canonical actin filaments, thus enhancing our molecular understanding of the dynamic space required for filament incorporation. We show that a deeper understanding of *Plasmodium* actin dynamics sheds light on the fundamental properties of the actin polymer across eukaryotes. Such findings provide insights into the structural space required for actin subunits to assemble in order to meet the diverse needs for filament dynamics of different cells, ranging from protozoan parasites through to classical mammalian systems.

## Materials and methods

### Ethics statement

All animal experiments were performed according to the German Animal Welfare Act (Tierschutzgesetz) and were approved by the responsible German authorities (Regierungspräsidium Karlsruhe, numbers G3/11, G-134/14, G-283/14).

### Computational methods

**Monomer MD simulation**

#### Preparation of structures

Coordinates of the *P*. *falciparum* actin 1 structure (PDB ID: 4CBU, 1.3 Å resolution) [22] were retrieved from the RCSB-protein data bank [75]. In this structure, the coordinates of chain G (gelsolin, water, and Ca^2+^) were deleted. The remaining structure, i.e., chain A, consisting of *P*. *falciparum* actin 1 (Q7-S366) complexed with ATP, Ca^2+^, and water molecules, was further prepared using the Protein Preparation Wizard of the Schrodinger software (version 2016r1). The co-crystallised Ca^2+^ ion was replaced with Mg^2+^ and co-crystallised waters were retained. The protonation of the titratable residues at pH 7 was predicted using PROPKA [76]. The missing loop region (G43-E50) residues were modeled using the Prime module of the Schrodinger software. Residue V11 was mutated to I11 in Maestro to give the *P*. *berghei* actin 1 sequence. Similarly, the structure of rabbit muscle alpha actin complexed with gelsolin (PDB ID: 1EQY) [77] was downloaded from the RCSB-PDB. The coordinates for the gelsolin chain were deleted and the remaining structure was prepared as described above for *Plasmodium* actin 1.

#### All-atom MD simulations

The structures were prepared for all-atom MD simulations using the tleap program in the AMBER MD package version 14 (http://ambermd.org/). GAFF and ff14SB force fields were used to assign parameters to the ligand and protein, respectively [78]. The parameters for ATP were retrieved from the AMBER parameter database (http://research.bmh.manchester.ac.uk/bryce/amber) [79]. Each system was solvated in a truncated octahedral box using the TIP3P water model [80]. An ionic strength of 50 mM was modelled by the addition of K^+^ and Cl^−^ ions, and the systems were neutralised with Na^+^ counterions.

The systems were then energy minimised as follows. Firstly, a restrained energy minimisation (force-constant = 100 kcal/mol Å^2^ on the protein and the ligand) with an initial 500 steps using steepest descent, followed by 500 steps using conjugate gradient minimisation, was performed. Then, a restraint-free all-atom energy minimisation was carried out for 1,500 steps using steepest descent, followed by 1,000 steps using conjugate gradient minimisation. Nonbonded interactions were cut off at 8 Å and a particle-mesh Ewald treatment was applied.

The energy minimised systems were gradually heated (0 to 298 K in 80 ps) using the canonical ensemble (NVT). These systems were next equilibrated in an isothermal–isobaric ensemble (NPT) at 298 K. Berendsen temperature coupling and a constant pressure of 1 atm with isotropic molecule-based scaling was used in the equilibration. All covalent bonds to hydrogen atoms were constrained using the SHAKE algorithm and a time step of 2 fs was used throughout the simulation [81]. All production simulations were performed in periodic boundary conditions (PBCs) in the NPT ensemble for a duration of 300 ns.

The MD trajectories were analyzed using the CPPTRAJ module of AMBER14 [82] and VMD (www.ks.uiuc.edu/Research/vmd/)[83]. Pymol (version 1.8.2.3) (https://pymol.org/) and VMD (version 1.9.2) were used for visualisation.

#### Filament construction and dynamics

In this study, we built a rabbit (Rb) actin (Uniprot Id: P68135) filament structure based on the model derived from cryo-electron microscopy data at 3.6 Å resolution (PDB ID: 5JLF) of 5 protomers [15]. We built a structure of a 15 protomer–long actin filament by superimposition of the first protomer onto the fourth protomer of the 5JLF structure to cover the twisted double strand with a length of a half pitch, which has 13.5 protomers. We built 15-mer actin filament models for wild-type *P*. *berghei* (Pb) (Uniprot Id: Q4Z1L3), the Pb H195T/G200S/E232A triple mutant, and the Pb H195T, G200S, E232A, and N41H single mutants, using the Rb actin filament as a template structure. We used Modeller 9v10 software to build these homology models.

To reduce the high computational cost and maintain calculation accuracy, we performed MD simulations with the MARTINI CG force field [84] with the GROMACS 5.0.7 package [85]. All seven actin filament structures were mapped to a CG model with MARTINI version 2.2. In order to maintain the secondary and tertiary structures of the actin protein during the CG MD simulation, intramolecular harmonic restraints were applied to the backbone atoms of the protein with an elastic force constant of 500 kJ.mol^−1^. nm^−2^ and a distance cutoff of 5–9 Å (lower and upper limits). The secondary structure information was generated using DSSP version 2002 (https://swift.cmbi.umcn.nl/gv/dssp/). After mapping, we constructed simulation systems for each structure, all of which were immersed in a saline solution with box dimensions of 15 nm × 15 nm × 54 nm. The saline solution consisted of MARTINI nonpolarisable water molecules with randomly placed Na^+^ and Cl^−^ ions to neutralise the system, and the salt concentration was set to 50 mM to mimic experimental conditions. We performed energy minimisation with the steepest descent algorithm for 10,000 steps to remove bad contacts and to build a reasonable starting structure. Subsequently, the temperature (300 K) and pressure (1 bar) of the system were equilibrated with a short MD simulation using a leap-frog algorithm with PBC and a 20 fs time step. A short equilibration CG MD simulation for 20 ns was performed in the NVT ensemble, followed by 20 ns of equilibration in the NPT ensemble with Berendsen pressure coupling. Finally, a 10 μs–long production MD simulation was performed with the NPT ensemble and Parrinello-Rahman pressure coupling. The trajectory of atomic positions and energies was saved every 1 ns. All systems were simulated at least in duplicate. We excluded the ADP cofactor and Mg^2+^ ion from CG MD simulations, as CG parameters for ADP, Mg^2+^ ion, and methylated histidine are not available. We used a standard histidine instead of methylated histidine (which was only present in Rb actin). The ADP cofactor and Mg^2+^ ion are buried in the protein. Methylated histidine points towards the surface of the protomer and does not have interactions with other residues/protomer. Therefore, we expect these omissions to have no/minimal impact on the overall architecture of the actin filament during CG MD simulations.

#### Computational analysis of filament inter-subunit properties

To analyse inter-subunit differences between canonical Rb, Pb, and Pb mutant actin filaments, we computed the number of lateral (i^th^ subunit with i+1^th^ subunit) and longitudinal (i^th^ subunit with i+2^th^ subunit) residue–residue contacts between each neigbouring subunit across the 15-mer filament (chains A to O) using a bead–bead contact threshold of 5 Å for each snapshot of all replica CG MD trajectories across each of the actin filament species. To eliminate possible artefacts arising from finite-size filament effects, the terminal four subunits at each end of the filament were excluded from the analysis. The mean number of residue–residue contacts across all mid-filament subunits (chains E-K), snapshots, and replica production trajectories for each species was then calculated in terms of a pairwise residue contact map (S6 Fig). The overall contact contribution of a given residue to its lateral or longitudinal subunit (mean residue–subunit contacts) was finally computed by integrating over the corresponding pairwise residue–residue contacts. Analyses were performed using the software packages VMD1.9 and MATLAB. Various architectural properties, such as the angle of rotation (alpha angle), the axial separation of the protomers (h), the protomers per pitch in the genetic helix, and the protomers per half pitch in the double helix [27], were also calculated (S3 Fig).

### Parasitology methods

#### Animals

For all experiments, female 4–6-wk-old NMRI (for parasite propagation, blood stage growth rate assays, and mosquito feeds) and C57Bl/6 (for transmission studies) mice were used (obtained from Janvier Laboratories). *A*. *stephensi* mosquitoes were reared and maintained by standard breeding methods.

#### Generation of chimeric or mutant parasite lines

Transfection vectors were generated using standard molecular biology techniques. For the generation of a recipient line, the endogenous *actin 1* gene containing silent restriction sites to facilitate subdomain exchanges were ordered from GeneArt (Invitrogen Corp) and cloned into the Pb238 vector [86,87] with BamHI and XbaI restriction sites. The actin 5′ untranslated region (UTR) was amplified using primers 1 and 2 and cloned into the vector using restriction enzymes SalI and XbaI (all enzymes purchased from New England Biolabs). The flanking *actin* 3′ UTR was amplified using primers 3 and 4 and cloned into the transfection vector via AvrII and KpnI restriction sites. The 3′ UTR of dihydrofolate synthase (*dhfs*) was cloned from another vector [87] into the downstream region of the actin ORF using BamHI and EcoRV restriction enzymes. Finally the positive-negative selection cassette *hdhfr-yfcu* (standing for human dihydrofolate reductase, yeast cytosine deaminase and uridyl phosphoribosyl transferase fusion enzyme) [88] replaced the original selection cassette using primers 5 and 6 and restriction enzymes EcoRV and AvrII. Linearisation of the construct was achieved through SalI and PmeI digestion, followed by DNA isolation using ethanol precipitation. Transfections were then performed as described previously [89,90] and positively selected using pyrimethamine (0.07 mg/mL) to yield a recipient line. All lines generated in this study were performed in the *P*. *berghei* ANKA background [91]. A clonal parasite from the transfection mixture of integrants and resistant wild-type was obtained by limiting dilution.

*Actin 1* ORF replacement constructs were generated using the same starting Pb238 vector. Two synthetic *actin* genes (one a codon modified *P*. *berghei actin 1* version, whereby the sequence was modified to result in the same protein sequence but with reduced homology, and the other *Oryctolagus cuniculus* alpha skeletal muscle actin) were ordered from GeneArt, as above. Subdomains were exchanged by restriction digestion (of engineered silent codon changes) and ligation between their original sequencing vectors as follows: subdomain 1a AatII and BamHI, subdomain 2 AatII and NruI, subdomain 3b AfeI and ClaI, subdomain 4 FseI and AfeI. These chimeras were cloned into an intermediate Pb238 replacement vector (containing the actin 5′ and 3′ UTR flanking the ORF, the 3′ UTR replaced the *dhfs* 3′ UTR using primers 7 and 8) via BamHI and XbaI restriction sites. The transfection construct was linearised with SalI and PmlI, transfected as above, and integration selected by negative selection using 5-fluorocytosine (1 mg/mL in drinking water) [92]. This rendered a line free of the selection cassette and contained the desired change in actin sequence. Blood stage parasites were monitored by smearing a drop of tail blood and staining with Giemsa solution (Merck). Calculation of blood stage growth rates were performed post-limiting dilution, assuming a single parasite was injected per positive mouse, as described previously [93].

For ABPs in the wild-type or PbS4Oc backgrounds, *Plasmodium* profilin, and coronin-mCherry constructs (full-length and actin binding mutant K349E,R350E), both under the sporozoite specific ‘up-regulated in infective sporozoites 3’ (*uis3*) promoter, were bulk prepared for transfection and linearised as previously described [37,38]. A C-terminally truncated coronin (expressing a construct with residues 1–388) was generated and amplified with primers 9 and 10, cloned into the nb3D+ vector using NotI and XbaI restriction sites, and linearised as above. mCherry-ADF2 under the *uis3* promoter was amplified using primers 11 and 12 and cloned into the starting Pb238 vector containing the *uis3* promoter and mCherry with NotI and SpeI restriction enzymes. This construct was linearised with NdeI. Transfections were performed and were selected with pyrimethamine as above.

For additional copy construct generation, the Pb238 vector containing homology arms for chromosome 12 [86,93] and the *actin* genes was reemployed. The *uis3* promoter was amplified with primers 13 and 14 and cloned into the vector using SacII and NotI enzymes. mCherry was amplified with primers 15 and 16 and cloned with restriction enzymes NotI and XbaI. The gene design also included a 10–amino acid alanine linker (AAAASRAAAA) in frame between the mCherry and actin ORFs. All chimeras were then cloned into Pb238 using XbaI and BamHI restriction sites. Note that this original vector already contained the downstream *dhfs* 3′ UTR and selection cassette. The transfection constructs were linearised with PvuI, transfected as above, integration was selected for with pyrimethamine and confirmed by genotyping, and clonal lines were obtained by limiting dilution.

In order to generate the endogenous reversibly switchable enhanced GFP (rseGFP) Formin 1 (PBANKA_1245300) tag, the PlasmoGEM system was used. The intermediate stage tagging vector PbGEM-int-309594 was ordered from PlasmoGEM. Also, the recombination vector GW R6K Emerald was used. The coding region of *rseGFP* (vector pQE31, a kind gift of Mhlanga lab) was amplified with primers 17 and 18 and cloned with SacII and ApaI into the GW R6K Emerald vector, replacing the coding region of Emerald with rseGFP. The ligated vector was transformed into *pir1* cells and the *rseGFP* sequenced. Using the Gateway reaction, the GW R6K vector and the intermediate vector PbGEM-int-309594 were combined and transformed into TSA cells. Essentially recommended protocols from the PlasmoGEM website were used (http://plasmogem.sanger.ac.uk/static/supporting_pdfs/Small_Scale_Recombineering_and_Gateway_protocol_WTAC_Mal_Exp_Gen_2013.pdf). The GW R6K rseGFP plasmid was supplied to the PlasmoGEM team and can be requested.

#### Small-scale ookinete culture

Mice were infected by intraperitoneal injection of frozen parasite stocks and were monitored until parasitemia reached approximately 2%. Fresh blood was acquired through cardiac puncture and approximately 20 million parasites transferred to a naïve recipient mouse. Three days post-transfer, exflagellation was assessed by incubation of a drop of tail blood for 10 min at 20°C. Exflagellation centres were observed by light microscopy (Carl Zeiss GmBH, Jena, Germany). In the event that >1 centre/field of view was observed, whole blood (approximately 1 mL) was mixed with 12 mL complete ookinete medium (RPMI-1640, 25 mM Hepes, 300 mg/L L-glutamine, 10 mg/L hypoxanthine, 50,000 units/L penicillin, 50 mg/L streptomycin, 2 g/L NaHCO_3_, 20.48 mg/L xanthurenic acid, 20% foetal bovine serum [FBS], pH 7.8) and incubated for 20 h at 19°C. Non-ookinete material was depleted by centrifugation on a 63% nycodenz cushion (in phosphate buffered saline [PBS], 1,000 rpm, no brake), and the ookinete mixture was pipetted from the interface and pelleted by centrifugation at 1,000 rpm for 8 min. The pellet was resuspended in 1 mL of FBS-free ookinete medium. For motility assays, 200 μL of ookinetes was briefly centrifuged to further concentrate the parasites and resuspended in 20 μL of medium. Approximately 4–6 μL of this concentrate was added to a microscope slide and sealed with a coverslip. Imaging was performed on a Zeiss Axiovert 200 M fluorescence microscope (Carl Zeiss GmBH, Jena, Germany) and images acquired every 20 s for 15 min (DIC exposure time 150 ms). Average speeds were calculated using the ImageJ Manual Tracking plug-in.

#### Infection of *A*. *stephensi* mosquitoes and characterisation of mosquito parasite stages

A donor mouse was infected by intraperitoneal injection of a frozen parasite stock. Four to five days postinfection, approximately 20 million parasites were transferred to two recipient naïve mice. Transmission ability was assessed by exflagellation observation using the procedure and criteria described above. Prior to feeding, mosquitoes were starved for 4–6 h. Infected recipient mice were anaesthetised with a ketamine/xylazine mixture (87.5 mg/kg ketamine, 12.5 mg/kg xylazine) and placed on top of the mosquito cage for approximately 30 min to allow for sufficient insect feeding. Infected mosquitoes were maintained at 21°C and 80% humidity until analysis.

For quantification of midgut oocyst numbers, 20–30 midguts were isolated by dissection on day 12 postinfection. Freshly isolated midguts were fixed in 1% NP-40 and subsequently stained with 0.1% mercurochrome in PBS for 30 min. Stained organs were mounted in a minimal amount of PBS, covered with a coverslip, and counted using a 10× objective on a Zeiss Axiovert 200 M fluorescence microscope. To assess salivary gland invasion rates, salivary glands and midguts of 20–30 mosquitoes were obtained on day 19 postinfection and transferred to 50 μL RMPI-1640 (supplemented with 50,000 units.l^−1^ penicillin and 50 mg.l^−1^ streptomycin) on ice. Organs were crushed for 1 min using a plastic pestle and typically diluted 1:2 for salivary gland homogenates or 1:5 for midgut preparations. Samples were then loaded into a Neubauer counting chamber and final counts normalised to sporozoites per mosquito. Sporozoite motility assays were performed on freshly dissected salivary glands, whereby organs were isolated and crushed, as above. Parasites were then purified from mosquito debris by centrifugation at 2,500*g* for 20 min on a 17% accudenz cushion [94]. Sporozoites at the interface were collected by pipetting, recentrifuged at maximum speed on a benchtop centrifuge, and activated by resuspension in 100 μL of 3% BSA in RPMI-1640 medium. This solution was transferred to a 96-well optical bottom plate (Nunc) and centrifuged at 1,000 rpm for 3 min to mediate parasite attachment to the plate bottom. Sporozoites were imaged using a 25× objective on a Zeiss Axiovert 200 M fluorescence microscope (DIC channel, 150-ms exposure time, frame rate: 1 frame/1.5 s). Average speeds were calculated using the ImageJ Manual Tracking plugin. Sporozoites were classed as continuously moving if parasites moved continuously for at least 75 s with no more than a 15 s halt in motility. Partially moving sporozoites were classed as such if these cells moved more than one parasite length but did not fulfill the criteria of continuous motility. Attached parasites were clearly attached to the surface but did not move more than one parasite length (or did not move productively at all). Parasites were considered to have paused if, in the case of a continuous or partial moving sporozoite, there was no discernible movement for three or more three frames (>4.5 s) or upon observation of a parasite briefly reversing. For localisation studies of additional copy parasite lines, parasites were imaged with a 63× objective using the same setup as above (mCherry channel λ_ex_ 546 nm, λ_em_ 575 nm, 25% filter, exposure 200 ms; DIC 150 ms). Comparison between parasite lines expressing *Plasmodium* actin from the UIS3 and actin promoters was performed using the mCherry channel (except using no extra filter and 300 ms exposure). Formin 1 localisation was assessed using dual lasers (405 nm and 488 nm) to switch the GFP to an active state and was imaged with a Nikon Ti series spinning disc confocal microscope (60× objective). For actin modulating compound response studies, after motility assay, imaging sporozoites were treated with either jasplakinolide or cytochalasin D (Sigma Aldrich), the solution mixed by gently pipetting, and the 96-well plate re-centrifuged. Imaging was performed as above. All images were processed in FIJI [95]. Parasite transmission studies back into naïve mice were performed as described previously [93]. Given the reduced numbers of salivary gland–resident parasites in the PbS4Oc line, 1,000 sporozoites were injected IV and thus had an appropriate control dose group.

#### Mammalian cell biology methods

*Actin* ORFs were amplified from starting vectors using primers 19 and 20 (for *Plasmodium actin 1* backbones) or 21 and 22 (for *O*. *cuniculus* beta actin backbones), digested with BamHI and XbaI, and cloned in frame with eGFP (with a 22–amino acid linker) in vector pEGFP-C1 (Clontech, Takara Bio Co., USA) using the same restriction enzymes. All constructs were confirmed by sequencing before transfection.

All media components were purchased from ThermoFisher (Gibco, USA). B16-F1 cells (ECACC 92101203) were maintained in DMEM, 10% FBS, and 2 mM L-Glutamine and Chinese Hamster Ovary cells (CHO-K1, ATCC CCL-61) were maintained in DMEM/F-12 supplemented with FBS and L-Glutamine, as above. Cells were plated one day prior to transfection such that the wells in a 24-well plate had a confluency of 70%–80%. Cells were transfected with 0.5–1 μg of plasmid DNA in duplicate in a 24-well plate using the Lipofectamine 2000 transfection reagent according to the manufacturers protocol (ThermoFisher). Twenty-one hours post-transfection, cells were lifted with trypsin-EDTA and approximately 15,000 B16-F1 cells or 30,000 CHO-K1 cells transferred to a new 24-well plate containing an acid-washed and protein-treated (for B16-F1 cells: 25 μg/mL laminin [Sigma-Aldrich]; for CHO-K1 cells: 50 μg/mL fibronectin [Sigma-Aldrich]) coverslip in 500 μL pre-warmed medium. Cells were allowed to settle for 3 h, were washed with 1 mL pre-warmed 1×PBS, and fixed with 500 μL 4% PFA, pH 7.4, for 15 min. Samples were washed three times with 1×PBS to remove fixative and subsequently incubated with 100 nM TRITC-phalloidin (Sigma) in 1×PBS. Staining mixture was removed, the samples washed three times, as before, and coverslips mounted in Mowiol 4–88 (Sigma). The mounted coverslips were allowed to set overnight at room temperature and stored at 4°C. For transfection and subsequent quantification, the experimenter was blinded to all sample identities. Images were acquired with the Zeiss Axiovert 200M (GFP channel λ_ex_ 450 nm, λ_em_ 515 nm, exposure 200 ms, rhodamine channel λ_ex_ 546 nm, λ_em_ 575 nm, exposure 200 ms). Approximately 30–50 cells were counted per transfection and all transfections performed at least three times (thus, >100 cells were characterised per condition). Cells that contained a GFP-positive actin network, as identified by phalloidin staining, were classed as positive for filament incorporation, whereas GFP expressing cells without observable co-localisation with phalloidin staining were classified as negative.

For B16-F1 cell motility assays, cell preparation and transfections were performed as above. After cells settled (approximately 24 h post-transfection), multiple GFP positive cells were imaged for 6–8 h in an environmentally controlled chamber of a Nikon Ti series spinning disc confocal microscope. Images were acquired using a 20× objective every 10 min and average cell speeds estimated from tracking the movement of the nucleus. Image processing and tracking were performed using Volocity (Perkin Elmer).

## Supporting information

S1 Fig Alignment of actin sequences from divergent species indicating regions of divergence. The secondary structure is indicated in grey, subdomains with black bars, and particular regions of interest are labelled with blue bars. Percentages in parentheses indicate sequence identity between *Plasmodium berghei* actin 1 and rabbit skeletal alpha actin for the regions specified.
(TIF)

S2 Fig Monomer breathing molecular dynamics simulation reveals only minor differences between actin species with different nucleotide states. (A) A schematic diagram illustrating subdomain (SD) positioning and the angles measured. Subdomains were defined as SD1: residues 6–33, 80–147, 334–349; SD2: residues 34–39, 52–69; SD3: residues 148–179, 273–333; and SD4: residues 180–219, 252–262. Scissor angle is the angle calculated as dot product between the two vectors drawn from centre of mass of C-alpha atoms of SD2 to SD1 residues and from centre of mass of C-alpha atoms of SD3 to SD4 residues. Dihedral angle is defined as dihedral angle between centre of mass of C-alpha atoms of SD2-SD1-SD3-SD4 residues. Clamp-mouth is the distance between C-alpha atoms of residue Q59 and E207. Clamp is the distance between C-alpha atoms of residue G15 and V157. Residue numbers mentioned here are for *Oryctolagus cuniculus* alpha skeletal muscle actin 1. (B) Relative subdomain orientations of each actin species in different nucleotide states are very similar between species. (C) Comparison of B-factor (as an indicator for residue flexibility) revealed a more flexible subdomain 4 region in the ATP-bound rabbit monomer. Underlying data can be found in S1 Data. SD, subdomain.
(TIF)

S3 Fig General structural parameters of average rotational angle between subunits (alpha angle) and subunits per half-pitch for the double helix computed from CG MD simulations correspond to previously published EM structures. α-angle values for Rabbit and *Plasmodium falciparum* actin filaments reported for cryo-EM structures are 166.9° (EMD-8162) and 167.5° (EMD-2572 and EMD-3805), respectively, and corresponding number of subunits per half-pitch for the double helix are 13.74 and 14.42, respectively. Results from our CG MD simulations sufficiently explained the difference of around 1 subunit per half-pitch for the double helix with 12.89 and 13.98 subunits for Rabbit and *P*. *berghei*, respectively. *P*. *berghei* subdomain 4 triple mutant (H195T, G200S, and E232A, see Fig 2) displays parameters (subunits per half-pitch for the double helix = 12.71) similar to rabbit actin (*Oryctolagus cuniculus*). The data presented here were obtained from stable trajectories (4–10 μs, based on filament RMSD values observed during CG MD simulation) of multiple CG MD simulations for each filament model. Analysis was performed on the middle chunk of filament—that is, the middle 7 subunits of filament structure, excluding 4 subunits on each terminal. The calculated α-angle values with standard deviation for Rabbit, *P*. *berghei*, and *P*. *berghei* HGE/TSA triple mutant are 166.04 ± 10.04, 167.12 ± 8.67, and 165.84 ± 10.69, respectively. The number of subunits per half-pitch for the double helix was calculated using average alpha angle value. Underlying data can be found in S1 Data. CG, coarse-grained; EM, electron microscopy; MD, molecular dynamics; RMSD, root-mean-square deviation.
(TIF)

S4 Fig A novel ‘gene-in marker-out’ screening strategy for the allelic replacement of the endogenous actin 1 gene with chimeras and mutants. (A) A linear construct containing a different 3′ UTR (*dhfs*) and positive/negative selection cassette (*yfcu*,*hdhfr*) is transfected into an unmodified line. Homologous recombination and positive selection with pyrimethamine renders a recipient line with a modified 3′ UTR region. (B) After obtaining a clone by limiting dilution, the recipient line is transfected with a linear construct containing homology regions in the 5′ and 3′ UTRs. Negative selection with 5-fluorocytosine selects for parasites having integrated the transfected construct and thereby lost the *yfcu*,*hdhfr* cassette. (C) The resulting transfected line therefore contains the desired chimera or mutation and leaves the UTRs with minimal changes. (D) Representative genotyping of transfected, recipient line (RL), and untransfected (U) lines. Relative primer positions, combinations (see S2 Table), and amplicon sizes are indicated, showing loss of the selection cassette and restoration of the locus. The open reading frame of each chimera or mutant was confirmed after transfection by sequencing. *dhfs*, dihydrofolate synthase; *hdhfr*, human dihydrofolate reductase; RL, recipient line; U, untransfected; UTR, untranslated region; *yfcu*, yeast cytosine deaminase and uridyl phosphoribosyl transferase.
(TIF)

S5 Fig Double mutations of subdomain 4 have no effect on parasite ability to penetrate mosquito salivary glands or motility. Thus, these three amino acids mutated in combination are required to phenocopy PbS4Oc, as reported in Fig 3. Broken lines in each graph represent the corresponding wild-type median values and solid line indicates PbS4Oc values (see main text for values and details of assays). In speed graph, red line indicates median value. ‘*n*’ is the total numbers of parasites counted per group. Underlying data can be found in S1 Data. HG/TS: H195T,G200S; HE/TA: H195T,E232A; GE/SA: G200S,E232A double mutants.
(TIF)

S6 Fig Heat maps of lateral and longitudinal subunit–subunit amino acid residue contact pairs normalised across the central monomers (monomers E–K; black indicates highest contact time for an amino acid contact) indicate enhanced contacts for F54 and Y189. Highest intensity contact pairs for HGE/TSA mutant are indicated with a red arrow. Underlying data can be found in S1 Data.
(TIF)

S7 Fig Generation of overexpression additional copy tagged actin binding proteins ADF2, profilin, and coronin (with mutants). (A) A generic representation of single crossover integration of the actin binding protein DNA construct in the modified actin background. (B) Final genomic arrangement of the integrated constructs. Relative primer positions, combinations (see S2 Table), and amplicon sizes are indicated. Please note that the C-terminally truncated coronin construct contained introns (+int), while full-length constructs were cDNA. (C) Representative genotyping of transfected (T) and untransfected (U) lines. Primer combinations are indicated above gel images and expected amplicon sizes below. +int, intron; ADF2, actin depolymerising factor 2; T, transfected; U, untransfected.
(TIF)

S8 Fig Generation and characterisation of additional copy actin chimeras and a C-terminally tagged formin 1. (A) Additional copy constructs were transfected and homology arms targeted integration into an intergenic locus in chromosome 12. Integrants were positively selected using pyrimethamine (selecting for the *hdhfr* cassette). (B) Final arrangement of relevant actin genes. Note that the chromosome 12 locus expresses a tagged actin construct in a stage-specific manner (Fig 4A). The endogenous actin 1 locus remains unmodified in these lines and non-tagged endogenous actin is thus also expressed. (C) Genotyping result of two transfected (trans) lines indicated correct integration, compared to an untransfected (U) control. (D) A modified PlasmoGEM construct was transfected and homology arms targeted integration into the terminal region of the open reading frame. Integrants were positively selected using pyrimethamine (selecting for the *hdhfr*/*yfcu* cassette). (E) The final integration state of C-terminally tagged formin 1. (F) Genotyping result of transfected parasites indicates correct integration within a mixed population. (G) Formin 1 has a distinct localisation to the apical tip of the parasite. Note the slight shift between channels due to parasite motility during image acquisition. eGFP, enhanced GFP; *hdhfr*, human dihydrofolate reductase; *rseGFP*, reversible switchable enhanced GFP; trans, transfected; U, untransfected; *yfcu*, yeast cytosine deaminase and uridyl phosphoribosyl transferase.
(TIF)

S9 Fig Comparison of relative expression levels of actin and UIS3 promoters. (A) Representative images of both parasite lines expressing mCherry *Plasmodium* actin from two different promoters. Scale bar: 5 μm. (B) mCherry signal was quantitated as described in the Materials and methods using a Fluorescence intensity. On average, the UIS3 promoter resulted in approximately 30% higher intensity (Mann–Whitney test, red line indicates median). Underlying data can be found in S1 Data. UIS3, Up-regulated in infective sporozoites 3.
(TIF)

S10 Fig Expression of divergent actin species in Chinese Hamster Ovary cells. (A) Representative images of CHO-K1 cells transfected with GFP-tagged rabbit or *Plasmodium* actin. Rabbit actin incorporates readily into filamentous structures of the cell, while parasite actin is not efficiently incorporated. (B) Quantification measured by percentage of cells positive for a GFP positive actin network. Values given as mean ± standard error of the mean of two independent experiments. Underlying data can be found in S1 Data. CHO, Chinese Hamster Ovary; GFP, green fluorescent protein.
(TIF)

S11 Fig Tentative model outlining the molecular basis for parasite pausing. Rapid motility relies on highly dynamic turnover of actin filaments. In the wild-type parasite, transient and short filaments (dark blue, small arrowhead) are built from monomers (light blue) at the parasite tip and are translocated to the rear by myosin. This retrograde flow results in a rearward direction of force, ultimately propelling the organism forward as it attaches on a substrate. At the rear, the filaments need to be rapidly disassembled (large arrowhead). This efficient turnover allows for relatively consistent sporozoite movement. Jasplakinolide treatment results in a collection of presumably longer filaments (small arrowhead) primarily at the front and rear of the parasite (large arrowheads). This increased stability leads to reduced filament recycling and thus the parasite stops moving. Mutations in actin subdomain 4 result in longer and more stable filaments, as in the wild type. At the parasite rear, a reduced rate of disassembly might cause a delay in filament recycling. This shift in equilibrium might cause a pause in motility until sufficient restoration allows the parasite to generate the required filament flow. Filament orientation is kept simple in this cartoon but might also change and lead to the observed rearward motion.
(TIF)

S1 Movie An example of a wild-type sporozoite moving on glass. (AVI)

S2 Movie An example of a PbS4Oc sporozoite moving on glass. Note the regular pauses and brief reversal of this parasite.
(AVI)

S1 Table Conservation status of residue position in *Oryctolagus cuniculus* alpha skeletal muscle actin and *Plasmodium berghei* actin 1. Rows for the mutated residues are coloured consistently with Fig 1. Analysis was done on multiple sequence alignment of all homologs searched in UNIREF90 database with sequence identity of >35% (Total 150 Proteins). It was observed that the interfaces are more divergent, with average conservation score of 0.42 and 0.31 for Rabbit and *P*. *berghei* actin, respectively (http://consurf.tau.ac.il/2016/).
(DOCX)

S2 Table Primers used in this study. (DOCX)

S1 Data Numerical data used to generate plots in figures. (XLSX)

## Abbreviations

ABP: actin binding protein
ADF: actin depolymerising factor
ADP: adenosine diphosphate
ATP: adenosine triphosphate
CG: coarse-grained
CHO: Chinese Hamster Ovary cells
dhfs: dihydrofolate synthase
D-loop: DNAse I-binding loop
EM: electron microscopy
F-actin: filamentous actin
FBS: foetal bovine serum
FP: fluorescent protein
G-actin: monomeric actin
GFP: green fluorescent protein
hdhfr: human dihydrofolate reductase
H-plug: hydrophobic plug
IDIP: Infectious Diseases Imaging Platform
IV: intravenous
MD: molecular dynamics
PBC: periodic boundary condition
PBS: phosphate buffered saline
Pi: inorganic phosphate
rseGFP: reversibly switchable enhanced green fluorescent protein
RMSD: root-mean-square deviation
UIS: up-regulated in infective sporozoites
UTR: untranslated region
yfcu: yeast cytosine deaminase and uridyl phosphoribosyl transferase
