# Supplementary figures and images for: Inter-subunit interactions drive divergent dynamics in mammalian and Plasmodium actin filaments
Source: PLoS Biol. 2018 Jul 16;16(7):e2005345. doi: 10.1371/journal.pbio.2005345 (PMC6055528; doi:10.1371/journal.pbio.2005345)

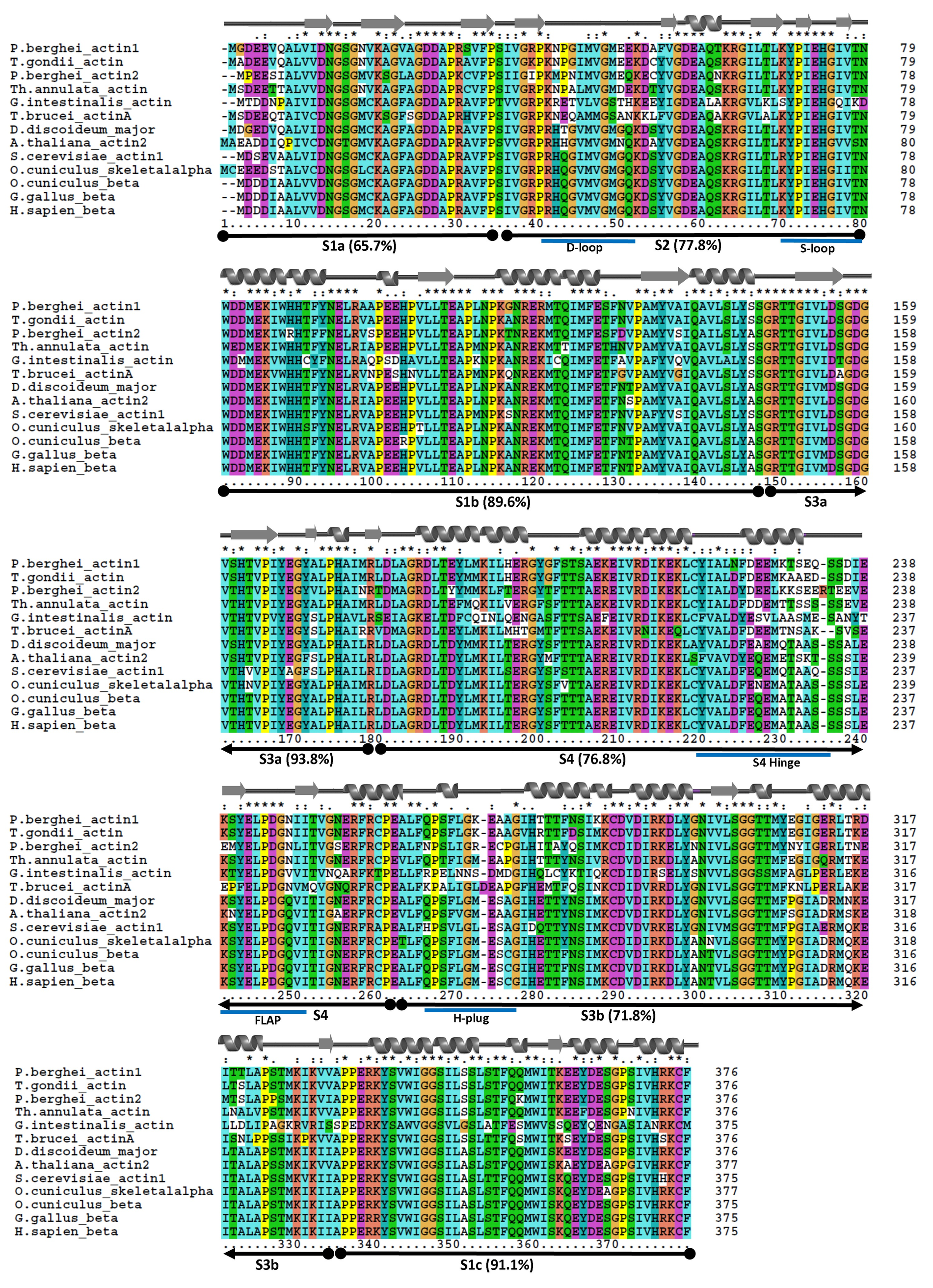

Supplement: S1 Fig — The secondary structure is indicated in grey, subdomains with black bars, and particular regions of interest are labelled with blue bars. Percentages in parentheses indicate sequence identity between Plasmodium berghei actin 1 and rabbit skeletal alpha actin for the regions specified. (TIF) [file pbio.2005345.s001.tif]

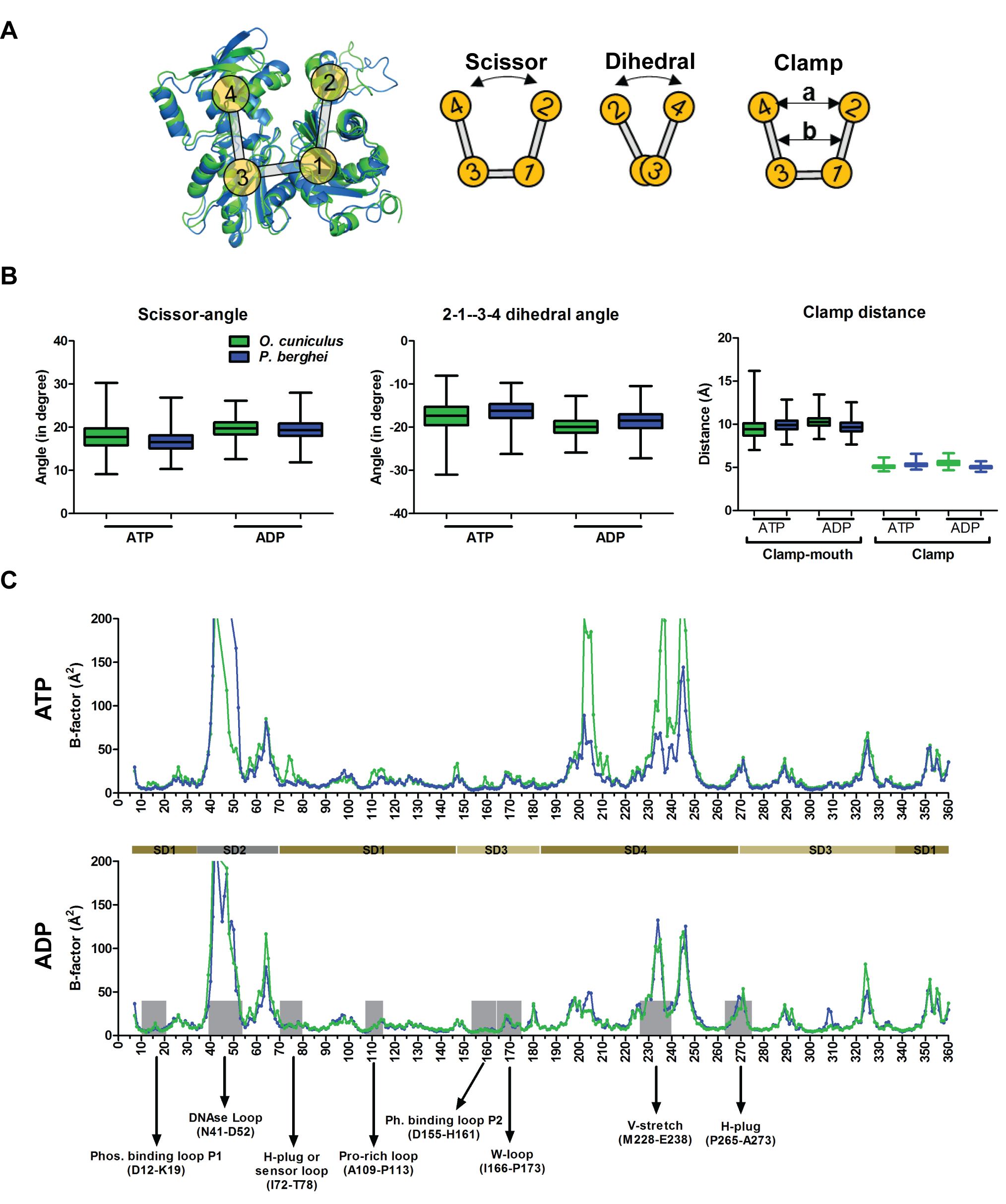

Supplement: S2 Fig — (A) A schematic diagram illustrating subdomain (SD) positioning and the angles measured. Subdomains were defined as SD1: residues 6–33, 80–147, 334–349; SD2: residues 34–39, 52–69; SD3: residues 148–179, 273–333; and SD4: residues 180–219, 252–262. Scissor angle is the angle calculated as dot product between the two vectors drawn from centre of mass of C-alpha atoms of SD2 to SD1 residues and from centre of mass of C-alpha atoms of SD3 to SD4 residues. Dihedral angle is defined as dihedral angle between centre of mass of C-alpha atoms of SD2-SD1-SD3-SD4 residues. Clamp-mouth is the distance between C-alpha atoms of residue Q59 and E207. Clamp is the distance between C-alpha atoms of residue G15 and V157. Residue numbers mentioned here are for Oryctolagus cuniculus alpha skeletal muscle actin 1. (B) Relative subdomain orientations of each actin species in different nucleotide states are very similar between species. (C) Comparison of B-factor (as an indicator for residue flexibility) revealed a more flexible subdomain 4 region in the ATP-bound rabbit monomer. Underlying data can be found in S1 Data. SD, subdomain. (TIF) [file pbio.2005345.s002.tif]

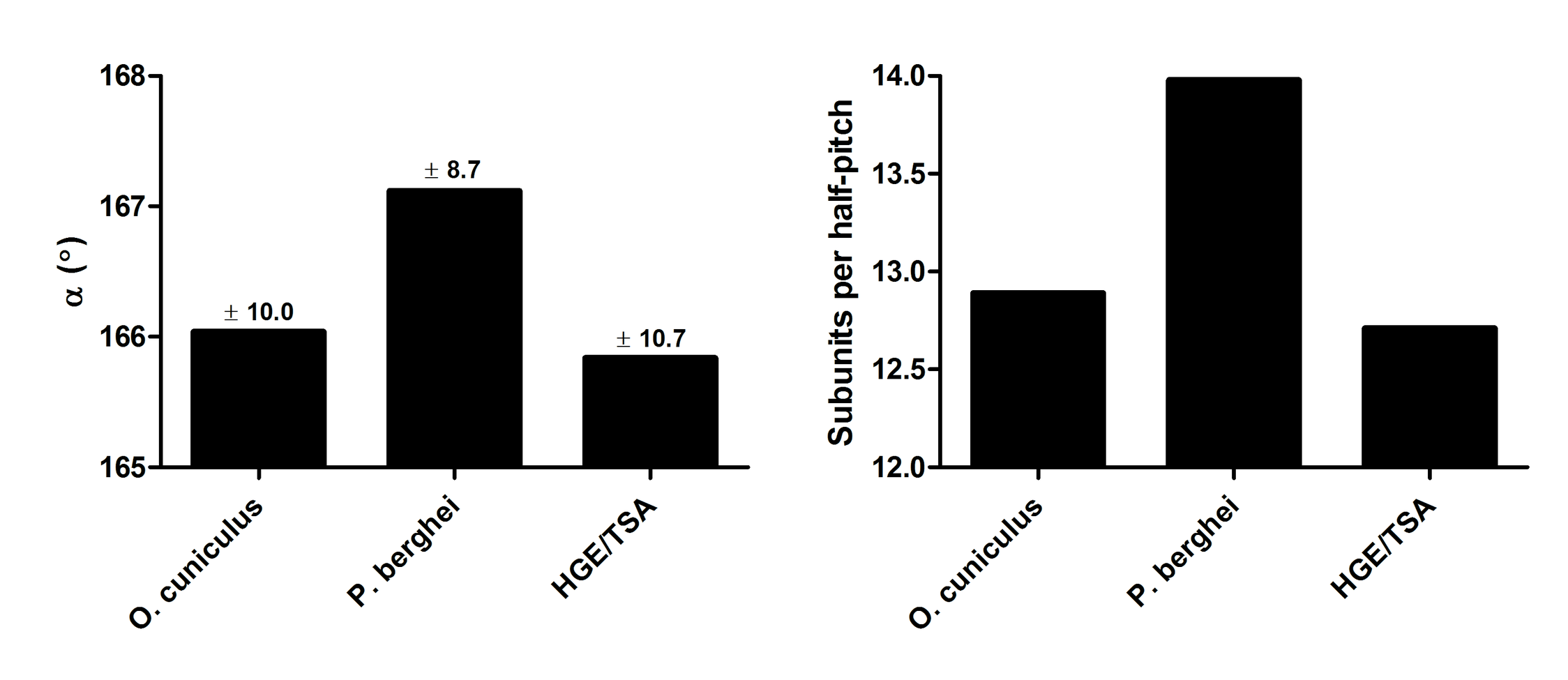

Supplement: S3 Fig — α-angle values for Rabbit and Plasmodium falciparum actin filaments reported for cryo-EM structures are 166.9° (EMD-8162) and 167.5° (EMD-2572 and EMD-3805), respectively, and corresponding number of subunits per half-pitch for the double helix are 13.74 and 14.42, respectively. Results from our CG MD simulations sufficiently explained the difference of around 1 subunit per half-pitch for the double helix with 12.89 and 13.98 subunits for Rabbit and P. berghei, respectively. P. berghei subdomain 4 triple mutant (H195T, G200S, and E232A, see Fig 2) displays parameters (subunits per half-pitch for the double helix = 12.71) similar to rabbit actin (Oryctolagus cuniculus). The data presented here were obtained from stable trajectories (4–10 μs, based on filament RMSD values observed during CG MD simulation) of multiple CG MD simulations for each filament model. Analysis was performed on the middle chunk of filament—that is, the middle 7 subunits of filament structure, excluding 4 subunits on each terminal. The calculated α-angle values with standard deviation for Rabbit, P. berghei, and P. berghei HGE/TSA triple mutant are 166.04 ± 10.04, 167.12 ± 8.67, and 165.84 ± 10.69, respectively. The number of subunits per half-pitch for the double helix was calculated using average alpha angle value. Underlying data can be found in S1 Data. CG, coarse-grained; EM, electron microscopy; MD, molecular dynamics; RMSD, root-mean-square deviation. (TIF) [file pbio.2005345.s003.tif]

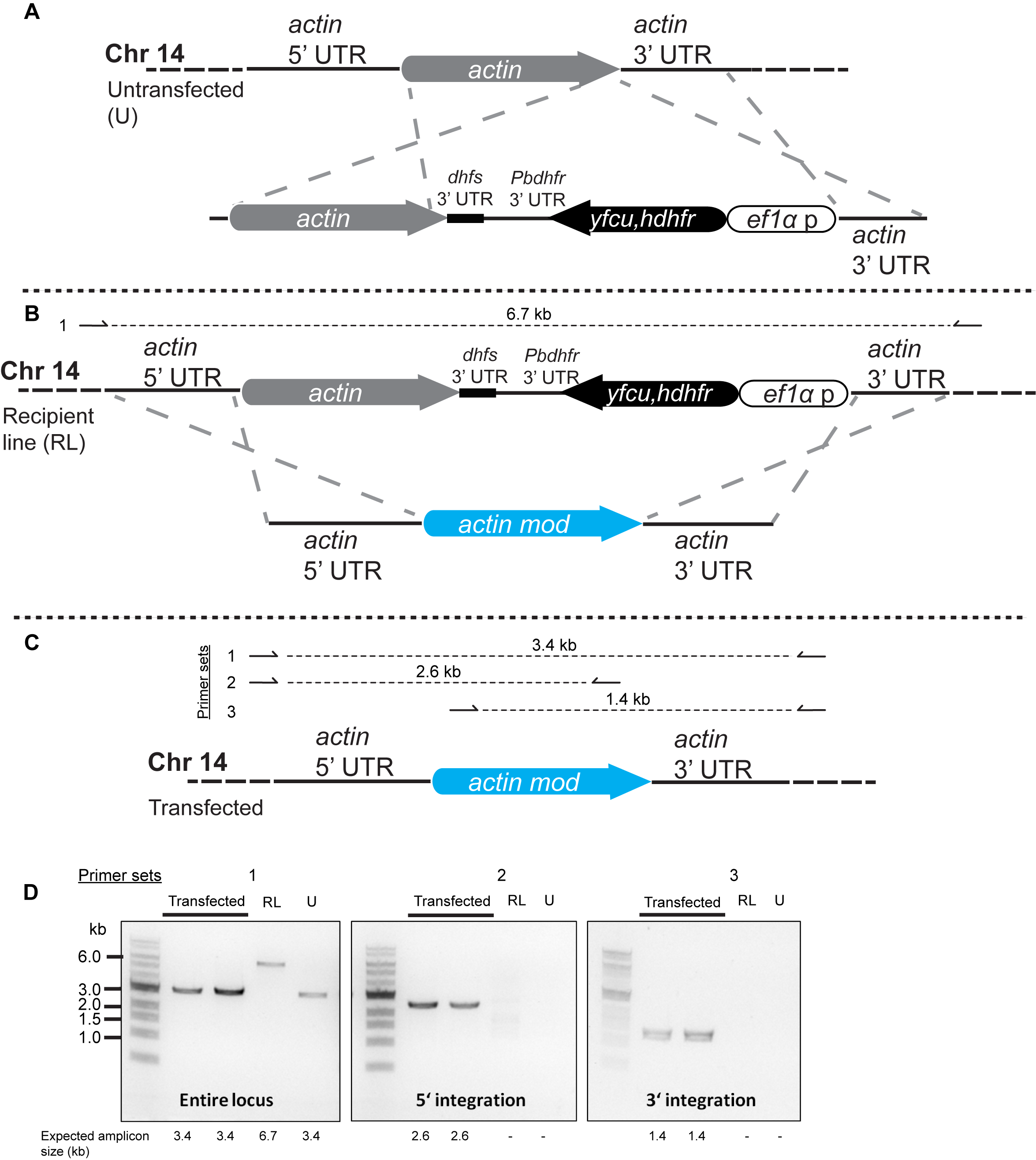

Supplement: S4 Fig — (A) A linear construct containing a different 3′ UTR (dhfs) and positive/negative selection cassette (yfcu,hdhfr) is transfected into an unmodified line. Homologous recombination and positive selection with pyrimethamine renders a recipient line with a modified 3′ UTR region. (B) After obtaining a clone by limiting dilution, the recipient line is transfected with a linear construct containing homology regions in the 5′ and 3′ UTRs. Negative selection with 5-fluorocytosine selects for parasites having integrated the transfected construct and thereby lost the yfcu,hdhfr cassette. (C) The resulting transfected line therefore contains the desired chimera or mutation and leaves the UTRs with minimal changes. (D) Representative genotyping of transfected, recipient line (RL), and untransfected (U) lines. Relative primer positions, combinations (see S2 Table), and amplicon sizes are indicated, showing loss of the selection cassette and restoration of the locus. The open reading frame of each chimera or mutant was confirmed after transfection by sequencing. dhfs, dihydrofolate synthase; hdhfr, human dihydrofolate reductase; RL, recipient line; U, untransfected; UTR, untranslated region; yfcu, yeast cytosine deaminase and uridyl phosphoribosyl transferase. (TIF) [file pbio.2005345.s004.tif]

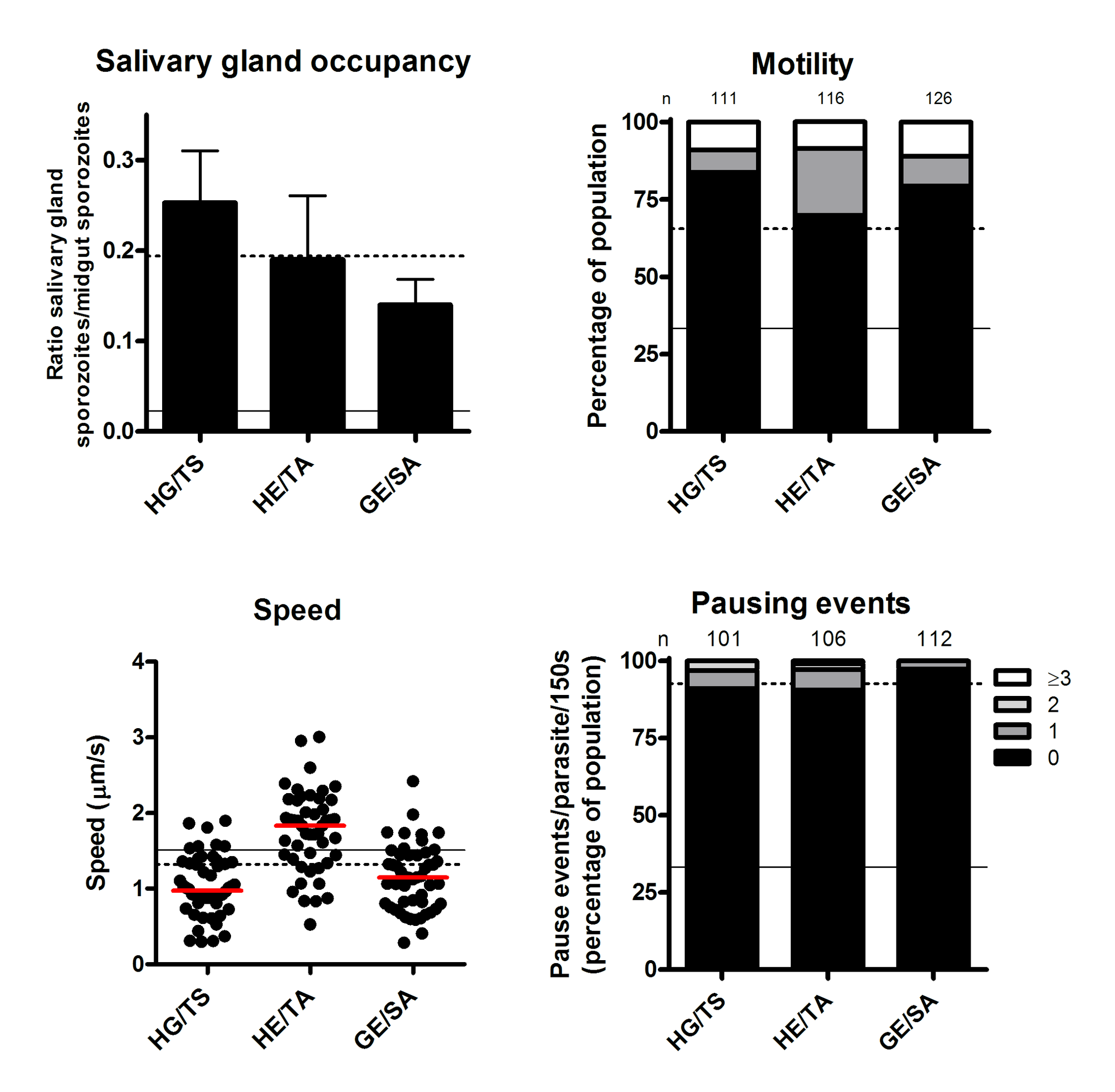

Supplement: S5 Fig — Thus, these three amino acids mutated in combination are required to phenocopy PbS4Oc, as reported in Fig 3. Broken lines in each graph represent the corresponding wild-type median values and solid line indicates PbS4Oc values (see main text for values and details of assays). In speed graph, red line indicates median value. ‘n’ is the total numbers of parasites counted per group. Underlying data can be found in S1 Data. HG/TS: H195T,G200S; HE/TA: H195T,E232A; GE/SA: G200S,E232A double mutants. (TIF) [file pbio.2005345.s005.tif]

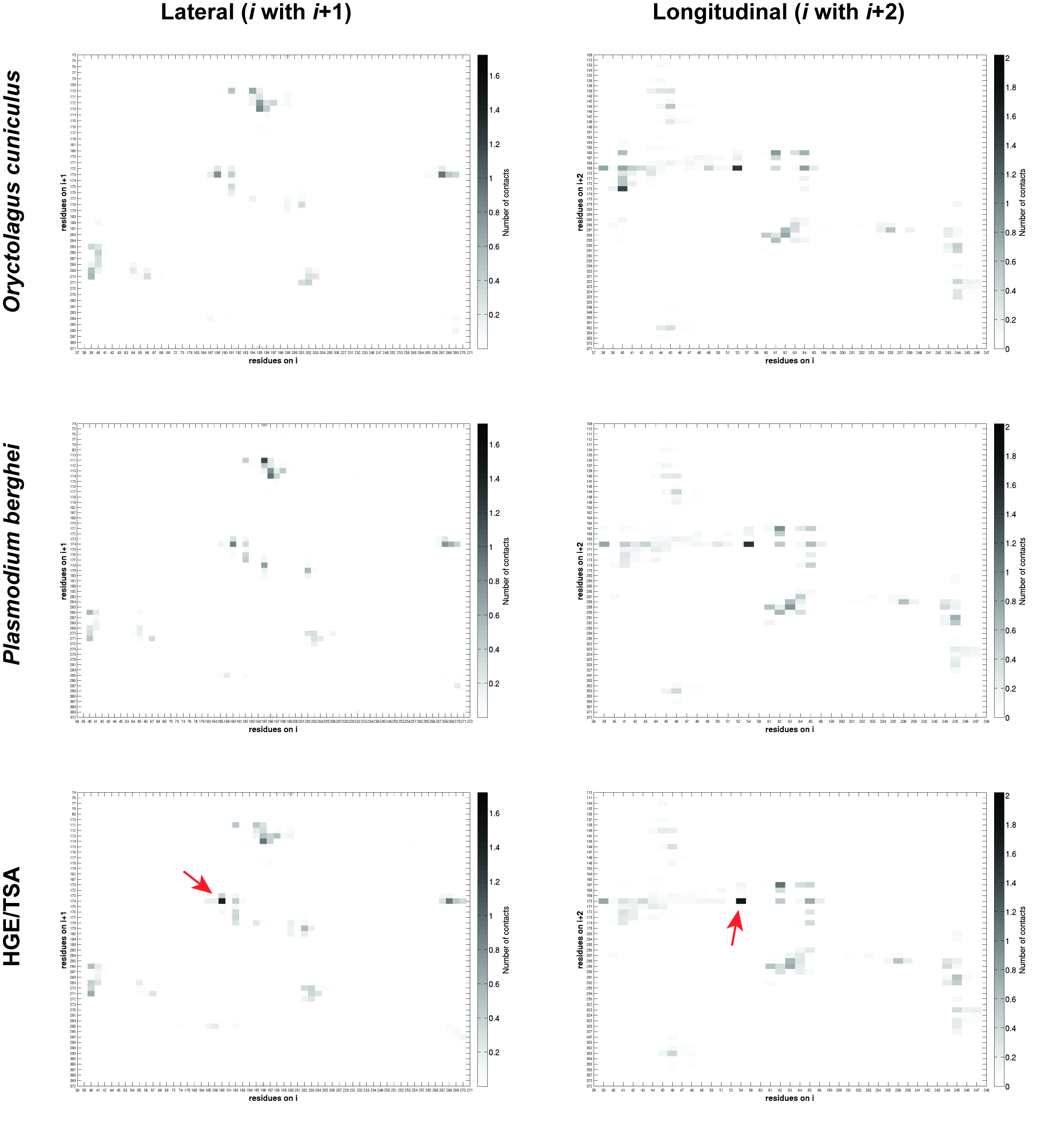

Supplement: S6 Fig — Highest intensity contact pairs for HGE/TSA mutant are indicated with a red arrow. Underlying data can be found in S1 Data. (TIF) [file pbio.2005345.s006.tif]

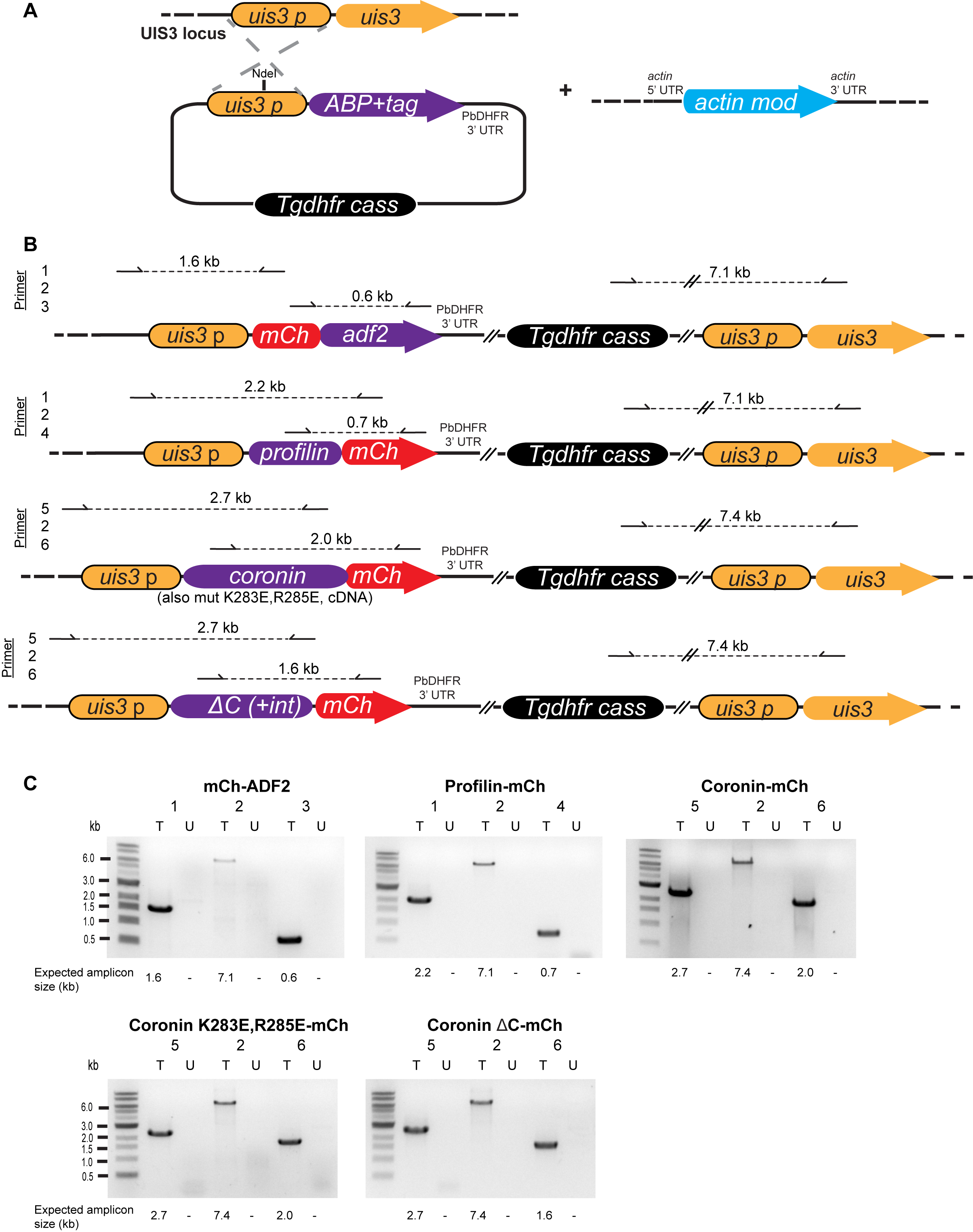

Supplement: S7 Fig — (A) A generic representation of single crossover integration of the actin binding protein DNA construct in the modified actin background. (B) Final genomic arrangement of the integrated constructs. Relative primer positions, combinations (see S2 Table), and amplicon sizes are indicated. Please note that the C-terminally truncated coronin construct contained introns (+int), while full-length constructs were cDNA. (C) Representative genotyping of transfected (T) and untransfected (U) lines. Primer combinations are indicated above gel images and expected amplicon sizes below. +int, intron; ADF2, actin depolymerising factor 2; T, transfected; U, untransfected. (TIF) [file pbio.2005345.s007.tif]

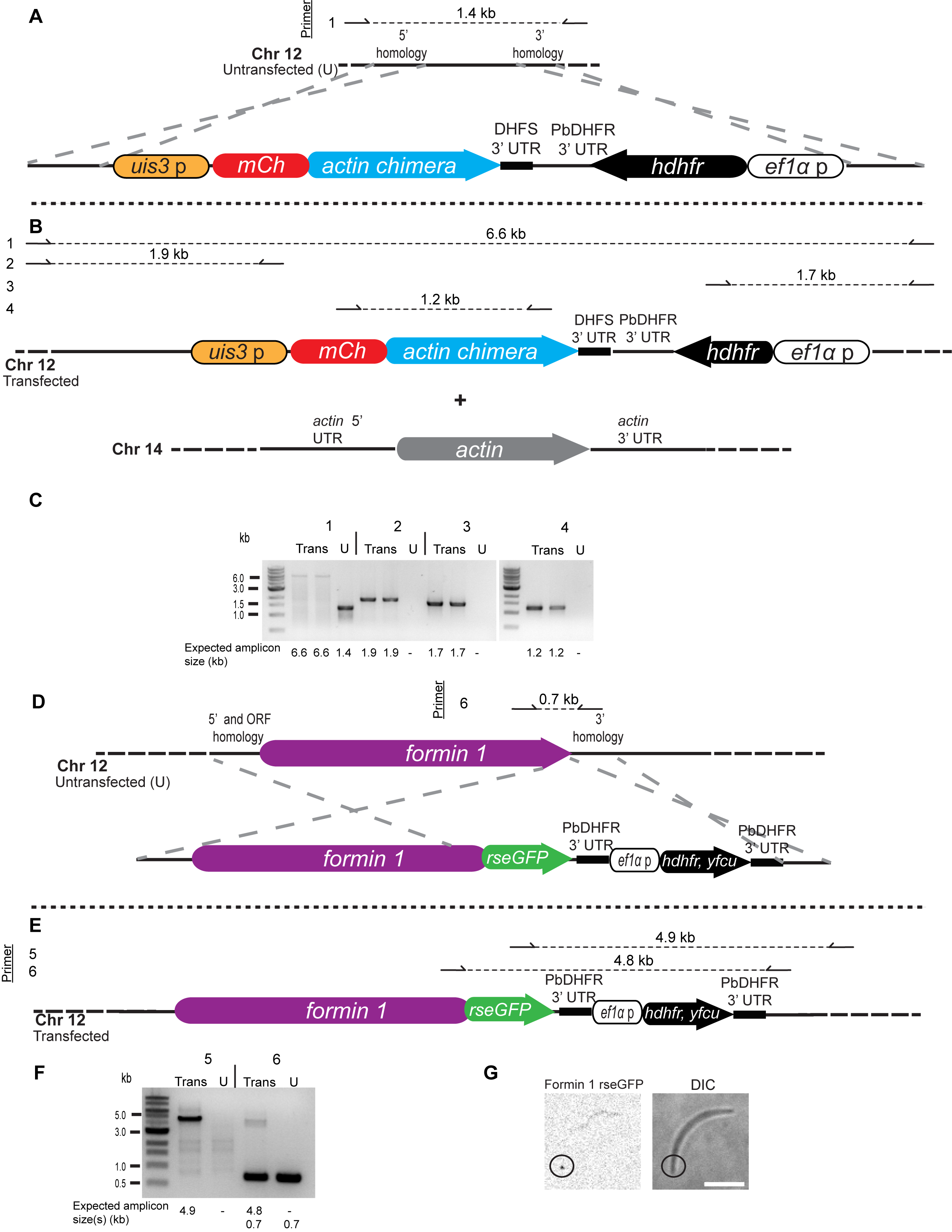

Supplement: S8 Fig — (A) Additional copy constructs were transfected and homology arms targeted integration into an intergenic locus in chromosome 12. Integrants were positively selected using pyrimethamine (selecting for the hdhfr cassette). (B) Final arrangement of relevant actin genes. Note that the chromosome 12 locus expresses a tagged actin construct in a stage-specific manner (Fig 4A). The endogenous actin 1 locus remains unmodified in these lines and non-tagged endogenous actin is thus also expressed. (C) Genotyping result of two transfected (trans) lines indicated correct integration, compared to an untransfected (U) control. (D) A modified PlasmoGEM construct was transfected and homology arms targeted integration into the terminal region of the open reading frame. Integrants were positively selected using pyrimethamine (selecting for the hdhfr/yfcu cassette). (E) The final integration state of C-terminally tagged formin 1. (F) Genotyping result of transfected parasites indicates correct integration within a mixed population. (G) Formin 1 has a distinct localisation to the apical tip of the parasite. Note the slight shift between channels due to parasite motility during image acquisition. eGFP, enhanced GFP; hdhfr, human dihydrofolate reductase; rseGFP, reversible switchable enhanced GFP; trans, transfected; U, untransfected; yfcu, yeast cytosine deaminase and uridyl phosphoribosyl transferase. (TIF) [file pbio.2005345.s008.tif]

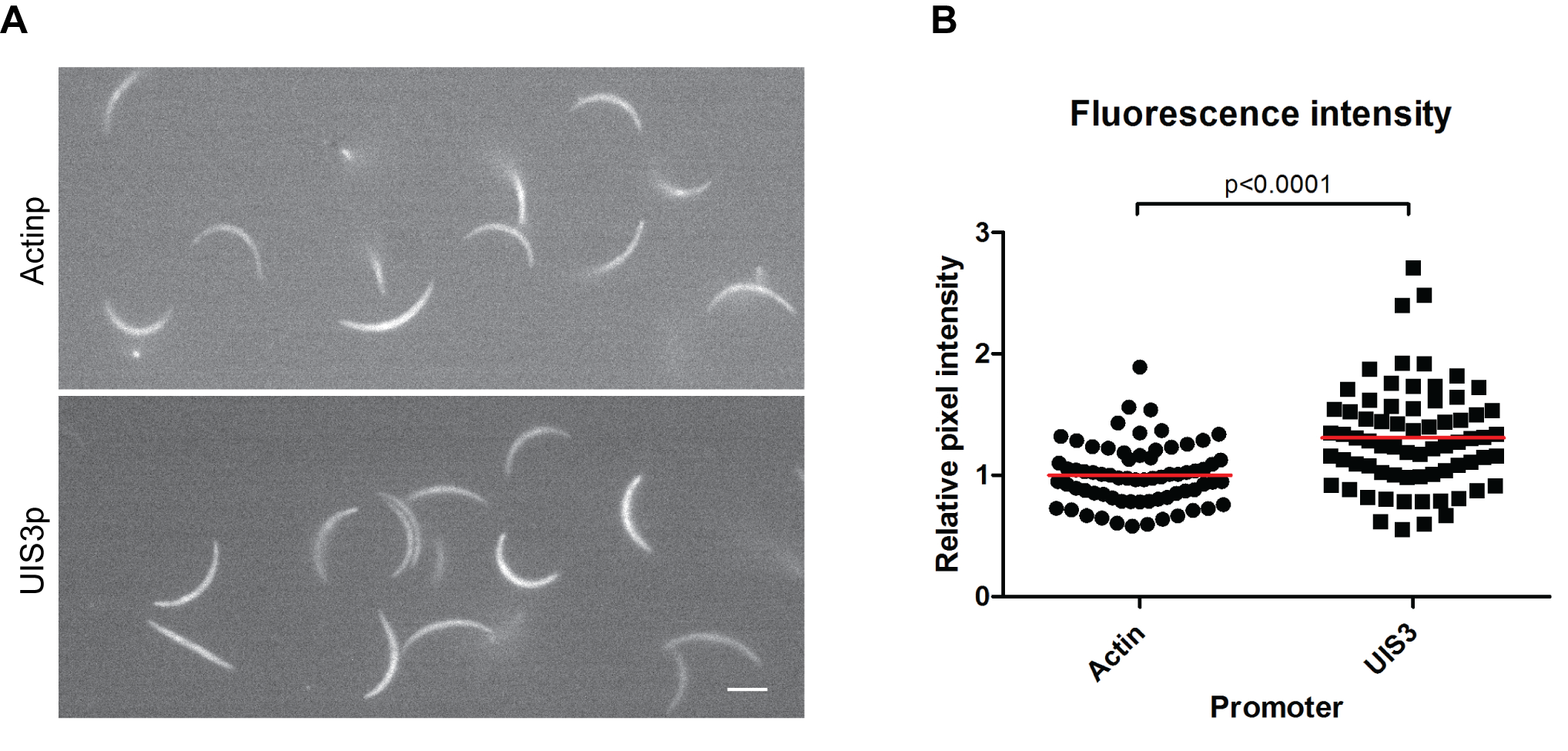

Supplement: S9 Fig — (A) Representative images of both parasite lines expressing mCherry Plasmodium actin from two different promoters. Scale bar: 5 μm. (B) mCherry signal was quantitated as described in the Materials and methods using a Fluorescence intensity. On average, the UIS3 promoter resulted in approximately 30% higher intensity (Mann–Whitney test, red line indicates median). Underlying data can be found in S1 Data. UIS3, Up-regulated in infective sporozoites 3. (TIF) [file pbio.2005345.s009.tif]

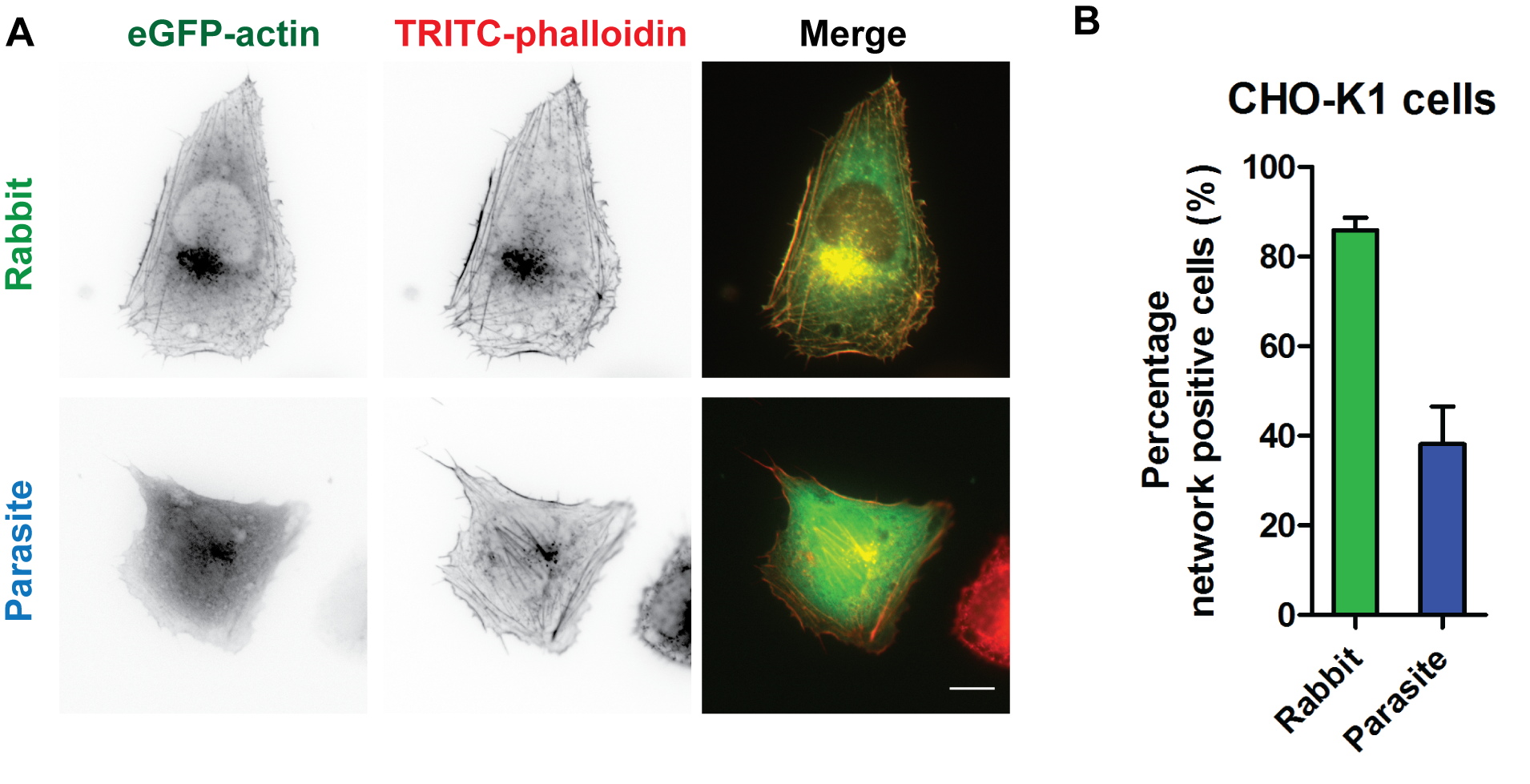

Supplement: S10 Fig — (A) Representative images of CHO-K1 cells transfected with GFP-tagged rabbit or Plasmodium actin. Rabbit actin incorporates readily into filamentous structures of the cell, while parasite actin is not efficiently incorporated. (B) Quantification measured by percentage of cells positive for a GFP positive actin network. Values given as mean ± standard error of the mean of two independent experiments. Underlying data can be found in S1 Data. CHO, Chinese Hamster Ovary; GFP, green fluorescent protein. (TIF) [file pbio.2005345.s010.tif]

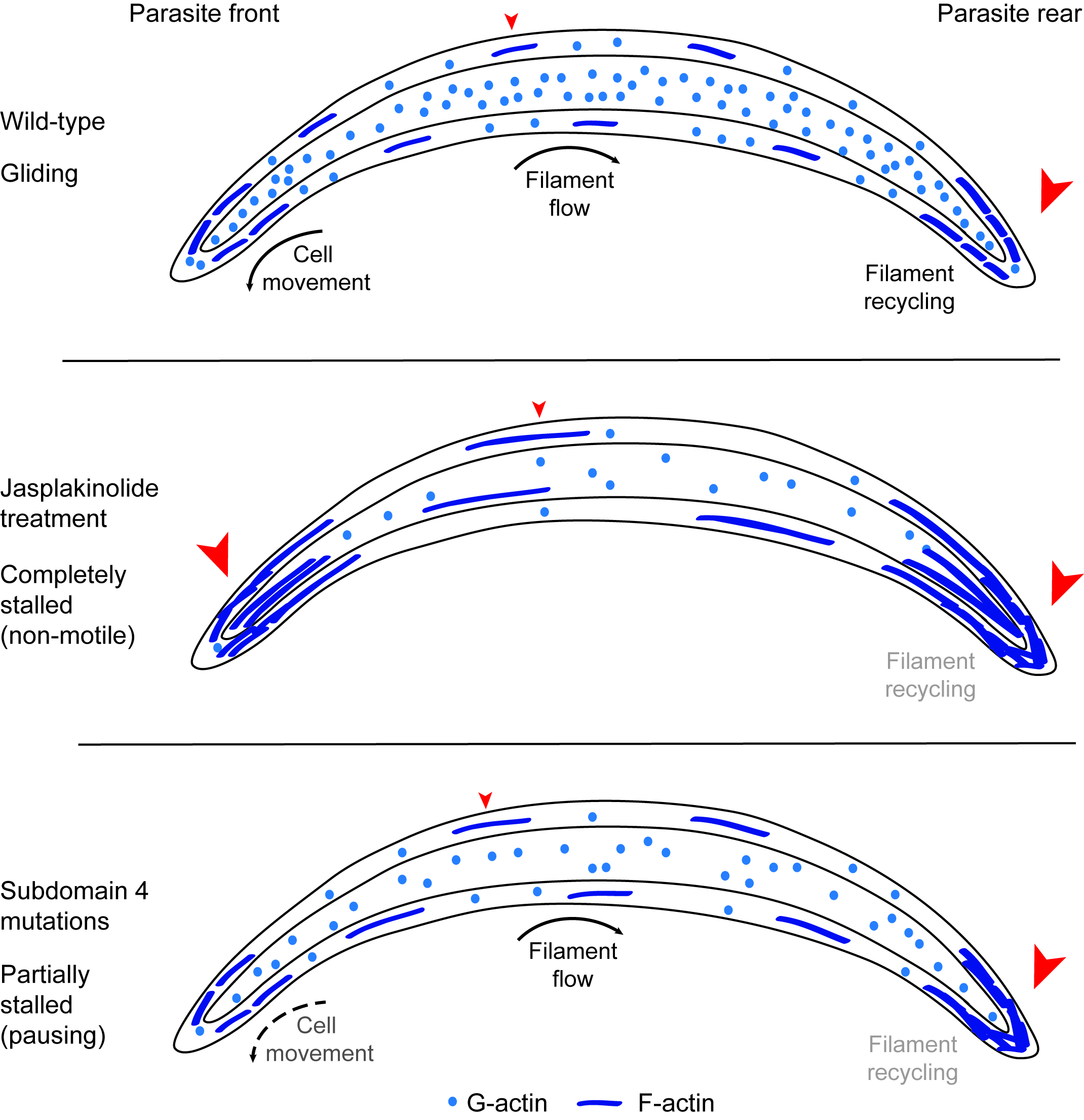

Supplement: S11 Fig — Rapid motility relies on highly dynamic turnover of actin filaments. In the wild-type parasite, transient and short filaments (dark blue, small arrowhead) are built from monomers (light blue) at the parasite tip and are translocated to the rear by myosin. This retrograde flow results in a rearward direction of force, ultimately propelling the organism forward as it attaches on a substrate. At the rear, the filaments need to be rapidly disassembled (large arrowhead). This efficient turnover allows for relatively consistent sporozoite movement. Jasplakinolide treatment results in a collection of presumably longer filaments (small arrowhead) primarily at the front and rear of the parasite (large arrowheads). This increased stability leads to reduced filament recycling and thus the parasite stops moving. Mutations in actin subdomain 4 result in longer and more stable filaments, as in the wild type. At the parasite rear, a reduced rate of disassembly might cause a delay in filament recycling. This shift in equilibrium might cause a pause in motility until sufficient restoration allows the parasite to generate the required filament flow. Filament orientation is kept simple in this cartoon but might also change and lead to the observed rearward motion. (TIF) [file pbio.2005345.s011.tif]
