# Supplementary material for: Inter-subunit interactions drive divergent dynamics in mammalian and Plasmodium actin filaments
Source: PLoS Biol. 2018 Jul 16;16(7):e2005345. doi: 10.1371/journal.pbio.2005345 (PMC6055528; doi:10.1371/journal.pbio.2005345)
Supplement: S1 Table — Rows for the mutated residues are coloured consistently with Fig 1. Analysis was done on multiple sequence alignment of all homologs searched in UNIREF90 database with sequence identity of >35% (Total 150 Proteins). It was observed that the interfaces are more divergent, with average conservation score of 0.42 and 0.31 for Rabbit and P. berghei actin, respectively (http://consurf.tau.ac.il/2016/). (DOCX) [file pbio.2005345.s014.docx]

| **Residues (*Oryctolagus cuniculus*)** | **Conservation score (normalized)** | **Scale^a^** | **MSA Data^b^** | **Residue variety^c^** |  | **Residue (*Plasmodium berghei*)** | **Conservation score (normalized)** | **Scale^a^** | **MSA Data^b^** | **Residue variety^c^** |
| --- | --- | --- | --- | --- | --- | --- | --- | --- | --- | --- |
| **R37** | 0.61 | 3 | 129/150 | N,S,C,F,K,V,W,Q,T,G,I,L,H,R,Y |  | **R38** | 0.67 | 3 | 129/150 | S,C,D,I,V,R,M,Y,T,F,L,H,E,K |
| **P38** | 0.61 | 3 | 127/150 | N,S,P,E,D,V,K,Q,R,G,A,L,H |  | **P39** | 0.32 | 4 | 129/150 | L,H,K,S,I,V,R,P,Q,A,N,T |
| **R39** | 0.46 | 4 | 127/150 | L,G,V,D,F,R,I,H,M,Y,Q,T,K,P,E,S |  | **K40** | 0.17 | 4 | 130/150 | S,C,T,Q,M,Y,L,H,E,V,I,G,D,N,R,P,A,K |
| **H40** | 1.75 | 1 | 125/150 | R,I,H,M,Y,Q,T,K,P,E,S,A,G,L,D,V,C,F,N |  | **N41** | 1.37 | 1 | 130/150 | K,V,I,G,D,N,P,R,L,F,H,E,S,C,T,W,Q,M,Y |
| **Q41** | 1.66 | 1 | 123/150 | F,D,V,N,G,L,A,K,E,P,S,H,I,R,Y,M,Q,T |  | **P42** | 2.34 | 1 | 129/150 | T,Q,M,Y,S,C,H,E,L,N,R,P,A,V,G,D,K |
| **G42** | 2.70 | 1 | 115/150 | N,V,D,C,G,A,P,E,S,K,T,Q,X,Y,M,R,I,H |  | **G43** | 1.43 | 1 | 120/150 | S,M,Q,Y,T,F,L,E,G,D,V,I,R,P,A,N,K |
| **V43** | 1.59 | 1 | 114/150 | C,V,N,L,A,G,W,K,E,P,S,M,I,R,T,Q |  | **I44** | 1.30 | 1 | 119/150 | W,T,A,Y,P,Q,M,I,V,G,S,K,E,L |
| **M44** | 0.34 | 4 | 111/150 | Q,T,A,H,I,L,M,Y,S,E,N,K,F,V,D |  | **M45** | 0.12 | 5 | 118/150 | K,I,V,D,G,N,A,P,R,L,F,E,S,T,Y,Q,M |
| **V45** | 1.38 | 1 | 99/150 | Y,M,I,R,T,K,S,E,P,L,A,G,F,V,D,N |  | **V46** | 1.48 | 1 | 111/150 | G,I,V,R,P,A,N,K,S,C,Q,M,T,F,L,H,E |
| **S52** | 1.54 | 1 | 132/150 | C,F,V,D,N,A,G,L,W,K,E,P,S,M,Y,I,R,T |  | **A53** | 1.63 | 1 | 135/150 | S,C,Q,Y,T,W,F,L,E,G,D,V,I,R,A,N,K |
| **Y53** | 0.00 | 5 | 136/150 | W,T,H,I,L,R,M,Y,C,F,V |  | **F54** | 0.35 | 4 | 137/150 | L,F,I,V,C,S,W,A,Y,P,M,Q |
| **S60** | 1.85 | 1 | 128/150 | T,Q,R,H,I,E,P,S,K,A,G,N,D,V,F,C |  | **T61** | 1.76 | 1 | 128/150 | C,S,Y,Q,T,F,E,H,D,G,V,I,A,R,P,N,K |
| **K61** | 1.04 | 2 | 132/150 | L,A,N,D,V,F,T,Q,M,R,I,H,S,P,E,K |  | **K62** | 0.73 | 3 | 133/150 | S,Y,Q,M,F,L,E,H,D,G,V,I,A,P,R,N,K |
| **R62** | 0.44 | 4 | 135/150 | G,A,L,N,D,V,C,T,Q,M,Y,R,I,H,S,E,P,K |  | **R63** | -0.48 | 6 | 132/150 | M,R,A,Y,N,G,S,D,V,I,H,E,K,F |
| **G63** | 1.56 | 1 | 135/150 | V,D,C,P,E,N,S,R,L,G,I,A,H,Y,T |  | **G64** | 0.99 | 2 | 136/150 | H,E,K,F,R,P,Q,A,T,N,S,G,D,I,V |
| **I64** | 0.90 | 2 | 133/150 | H,I,R,M,Y,Q,T,K,E,P,S,G,A,L,F,D,V,N |  | **I65** | 1.13 | 1 | 134/150 | G,V,I,A,R,P,N,F,L,E,H,S,Y,Q,M,T |
| **L110** | -0.31 | 6 | 148/150 | N,S,C,F,V,W,T,I,H,L,A,R,M |  | **L111** | 0.03 | 5 | 147/150 | H,E,K,F,L,P,Q,R,M,Y,N,S,I,V |
| **N111** | -1.01 | 8 | 148/150 | T,L,A,E,N,S,C,F |  | **N112** | -0.95 | 8 | 147/150 | F,S,G,C,I,Q,A,Y,T,N |
| **P112** | -0.46 | 6 | 148/150 | T,Q,S,P,A,I |  | **P113** | 0.08 | 5 | 146/150 | L,S,C,D,I,Q,R,P,A,N,T |
| **K113** | 0.37 | 4 | 147/150 | T,Q,M,I,A,H,L,R,P,N,E,S,F,K,V |  | **K114** | 0.55 | 3 | 145/150 | L,E,H,K,S,I,V,Y,A,R,P,Q,M,T,N |
| **V139** | -0.75 | 7 | 150/150 | Q,T,R,L,A,I,G,H,M,V,C,K |  | **V140** | -0.54 | 7 | 149/150 | H,L,F,X,T,Y,A,M,R,I,V,C,S |
| **Y143** | -0.90 | 8 | 150/150 | A,H,C,L,F,Y,T |  | **Y144** | -0.82 | 8 | 149/150 | H,F,W,M,Q,A,Y,I,S,C |
| **Y166** | 0.47 | 4 | 149/150 | V,F,K,S,N,L,A,H,I,Y,W,T |  | **Y167** | -0.17 | 6 | 149/150 | F,L,H,S,C,V,I,Q,Y,A,W |
| **E167** | -0.44 | 6 | 149/150 | R,D,K,Q,S,E,N |  | **E168** | -0.30 | 6 | 149/150 | Q,Y,A,N,T,D,E,K,L |
| **G168** | -1.05 | 8 | 150/150 | E,S,T,R,A,G |  | **G169** | -0.90 | 8 | 150/150 | W,E,A,S,G,F |
| **Y169** | 0.02 | 5 | 150/150 | Q,T,R,G,A,H,L,Y,N,S,V,K,C,F |  | **Y170** | 0.08 | 5 | 150/150 | T,Q,Y,S,C,H,E,L,F,N,R,A,V,I,D,K |
| **A170** | 0.73 | 3 | 150/150 | V,C,N,S,P,G,L,I,A,Y,Q,T |  | **A171** | 0.28 | 4 | 150/150 | L,K,I,V,G,S,C,N,T,P,Q,A,Y |
| **L171** | 0.09 | 5 | 150/150 | K,F,V,E,M,H,A,L,I,Q |  | **L172** | -0.49 | 7 | 150/150 | V,I,A,Q,P,M,L,F |
| **P172** | 1.31 | 1 | 150/150 | N,P,S,D,K,T,Q,Y,R,L,G,I,A,H |  | **P173** | 1.10 | 2 | 150/150 | I,G,S,C,D,N,T,P,R,Q,Y,A,L,K,H |
| **H173** | -0.38 | 6 | 148/150 | N,V,D,G,A,P,E,S,K,T,X,Q,Y,R,I,H |  | **H174** | -0.67 | 7 | 150/150 | Y,A,Q,P,R,T,N,C,D,G,S,E,H,K,L |
| **R177** | -0.58 | 7 | 150/150 | S,N,K,C,Q,T,R,L,H,A |  | **R178** | -0.52 | 7 | 150/150 | F,L,E,H,K,C,S,I,Q,R,W,T,N |
| **L178** | 0.58 | 3 | 150/150 | V,F,S,M,R,A,G,L,I,H,T,Q |  | **L179** | 0.19 | 4 | 150/150 | F,L,H,S,I,V,Q,M,R,N,T |
| **D187** | 1.82 | 1 | 150/150 | C,D,V,N,L,A,W,K,S,E,H,I,R,M,Y,Q,T |  | **E188** | 2.81 | 1 | 150/150 | K,I,D,G,N,A,R,L,F,E,H,C,S,T,Y,M,Q |
| **Y188** | 0.41 | 4 | 150/150 | F,E,N,S,L,I,A,H,R,Y,W,Q |  | **Y189** | 0.28 | 4 | 150/150 | N,W,R,Q,Y,A,S,H,E,L,F |
| **M190** | 0.22 | 4 | 150/150 | M,R,L,G,A,I,T,Q,W,V,D,C,K,N,S |  | **M191** | 0.40 | 4 | 150/150 | E,H,K,L,A,M,Q,R,W,T,N,G,S,V,I |
| **K191** | 1.20 | 1 | 150/150 | V,D,F,N,L,A,K,S,E,M,Y,R,I,H,T,Q |  | **K192** | 0.75 | 3 | 150/150 | N,A,R,D,G,K,T,Y,Q,M,C,S,E,H,L,F |
| **L193** | -1.00 | 8 | 150/150 | I,A,F,L,M,T |  | **L194** | -1.10 | 8 | 150/150 | M,L,I |
| **T194** | 1.29 | 1 | 150/150 | K,S,E,P,M,Y,I,H,R,T,Q,C,F,V,D,N,A,G,L |  | **H195** | 0.77 | 3 | 150/150 | F,L,H,K,S,G,D,M,R,Q,A,Y,N,T |
| **E195** | 0.23 | 4 | 149/150 | R,L,G,A,H,Q,T,D,K,E,N,S |  | **E196** | 0.42 | 4 | 148/150 | D,S,G,A,Y,M,Q,R,T,F,L,E,K |
| **R196** | 0.47 | 4 | 148/150 | G,A,L,H,R,T,Q,K,C,D,N,S,E |  | **R197** | 0.04 | 5 | 148/150 | E,H,K,L,A,Q,R,N,T,D,G,S,I,V |
| **Y198** | 1.57 | 1 | 144/150 | K,P,E,S,H,I,R,Y,Q,T,F,C,V,N,A,L |  | **Y199** | 1.06 | 2 | 147/150 | I,V,S,C,N,T,P,Q,R,Y,L,F,K,H,E |
| **S199** | 1.24 | 1 | 140/150 | P,S,K,T,Q,M,Y,R,H,I,N,V,D,F,G,A,L |  | **G200** | 1.03 | 2 | 143/150 | L,F,H,C,S,W,T,Y,Q,K,V,I,D,G,N,A,R,P |
| **F200** | -0.16 | 5 | 140/150 | F,D,V,N,S,M,Y,I,L,T |  | **F201** | -0.09 | 5 | 144/150 | I,V,S,Y,P,M,L,F |
| **V201** | 0.79 | 3 | 140/150 | R,H,I,M,Y,Q,T,K,P,E,S,L,G,A,V,D,F,N |  | **S202** | 1.01 | 2 | 136/150 | V,I,D,N,A,R,P,K,C,S,T,Y,Q,L,F,E,H |
| **T202** | -0.07 | 5 | 131/150 | T,Q,Y,I,H,R,P,E,S,K,L,A,N,F,C,V |  | **T203** | -0.32 | 6 | 145/150 | K,H,E,N,T,Q,P,R,A,I,V,S,G,C |
| **A204** | 0.16 | 5 | 147/150 | G,A,L,V,D,F,C,N,R,H,I,Y,M,Q,T,K,E,S |  | **A205** | 0.17 | 4 | 147/150 | D,G,V,I,A,R,N,C,S,Y,Q,M,T,F,L,E,H |
| **E205** | -0.25 | 6 | 148/150 | V,D,K,N,S,E,P,R,A,H,G,M,Q,T |  | **E206** | -0.47 | 6 | 147/150 | N,T,Y,A,M,R,Q,I,D,S,G,K,E,H |
| **L242** | -1.15 | 8 | 149/150 | V,C,L,G,M,T |  | **L243** | -1.11 | 8 | 145/150 | M,A,T,N,K,L |
| **P243** | -1.02 | 8 | 148/150 | I,A,D,V,E,S,P |  | **P244** | -0.68 | 7 | 146/150 | K,C,S,G,T,A,R,Q,P |
| **D244** | -1.14 | 8 | 148/150 | K,D,N,S,L,H,Y,T |  | **D245** | -1.11 | 8 | 146/150 | R,Q,P,A,Y,K,D |
| **S265** | 0.41 | 4 | 146/150 | E,N,P,S,K,C,D,Q,T,H,G,A,R,M,Y |  | **S266** | 0.42 | 4 | 144/150 | T,M,Q,C,S,E,H,L,F,N,A,P,V,D,G,K |
| **F266** | 0.72 | 3 | 145/150 | V,K,F,N,E,M,R,A,I,H,L,T,Q,X |  | **F267** | 1.10 | 2 | 143/150 | H,E,L,F,X,T,M,Q,Y,C,K,N,R,A,I,V,D |
| **I267** | 1.12 | 2 | 146/150 | R,I,H,Y,M,S,P,W,G,A,L,N,D,V,F,C |  | **L268** | 0.67 | 3 | 144/150 | V,I,S,G,C,T,N,M,Q,Y,A,L,F |
| **G268** | -0.40 | 6 | 147/150 | M,H,A,G,N,S,E,K,C,D |  | **G269** | 0.06 | 5 | 144/150 | V,D,S,G,N,A,P,M,Q,L,F,K,E |
| **M269** | 1.70 | 1 | 147/150 | G,L,W,F,C,D,V,N,H,I,R,Y,M,Q,T,K,P,E,S |  | **K270** | 1.02 | 2 | 144/150 | I,V,D,N,A,P,R,K,C,S,T,Y,M,Q,L,F,E,H |
| **E270** | 0.08 | 5 | 146/150 | N,S,P,E,D,K,Q,T,R,H,L,G,M |  | **E271** | -0.18 | 6 | 144/150 | E,H,Y,R,M,P,Q,N,T,D,G,S |
| **D286** | -0.32 | 6 | 145/150 | T,A,H,R,S,E,N,P,D,V |  | **D287** | -0.71 | 7 | 143/150 | Y,A,P,Q,D,G,S,K,E,H |
| **I287** | 2.55 | 1 | 145/150 | K,S,P,E,R,I,Y,M,Q,T,D,V,N,A,G,L |  | **V288** | 0.98 | 2 | 142/150 | G,S,D,V,I,M,P,Q,R,A,Y,T,L,E,K |
| **D288** | -0.43 | 6 | 145/150 | T,Y,G,A,R,E,N,S,C,D |  | **D289** | -0.55 | 7 | 143/150 | K,E,H,V,D,S,G,N,Y,A,R |
| **I289** | 0.36 | 4 | 145/150 | T,Y,M,H,L,A,I,N,S,V,F |  | **I290** | -0.06 | 5 | 142/150 | M,Q,A,N,T,S,C,V,I,E,F,L |
| **R290** | -0.78 | 7 | 145/150 | W,Q,L,H,R,S,K,C |  | **R291** | -0.87 | 8 | 142/150 | H,K,L,Y,R,Q,T,N,S |
| **K291** | 1.18 | 1 | 145/150 | D,K,C,N,S,P,E,M,R,A,L,T,Q |  | **K292** | 1.03 | 2 | 142/150 | D,S,G,N,T,A,Q,P,R,L,K,E,H |
| **P322** | 0.16 | 5 | 137/150 | T,Q,M,L,G,A,E,N,S,P,K,V |  | **P323** | 0.72 | 3 | 134/150 | F,L,E,G,S,C,D,I,V,M,P,Q,A,N |
| **Average** | **0.42** | **3.97** |  |  |  |  | **0.31** | **4.27** |  |  |

**Note:**

a: Representing the scale of normalized conservation scores (9 – conserved, 1 - variable).

b: The number of aligned sequences having an amino acid (non-gapped) from the overall number of sequences at each position.

c: The residues variety at each position of the multiple sequence alignment.
