# Supplementary material for: Inter-subunit interactions drive divergent dynamics in mammalian and Plasmodium actin filaments
Source: PLoS Biol. 2018 Jul 16;16(7):e2005345. doi: 10.1371/journal.pbio.2005345 (PMC6055528; doi:10.1371/journal.pbio.2005345)
Supplement: S2 Table — (DOCX) [file pbio.2005345.s015.docx]

| ***Plasmid generation*** | |  |  |  |
| --- | --- | --- | --- | --- |
| **Primer no.** | **Primer name** | **Sequence (5'-3')** | **Restriction site** | **Used for** |
| 1 | Actin 5' UTR F | ACGCGTCGACATAAATAGGGATATGATAAAATAAGAATTTAGAAATTATGAAAATAC | SalI | Actin 5' UTR cloning into vector Pb238 |
| 2 | Actin 5' UTR R | GCTCTAGATTTAATTTTTTTTTTAAGTATATGAGTATATATATGTGTGTAAAAATTTATATTAAATATGC | XbaI | Actin 5' UTR cloning into vector Pb238 |
| 3 | Actin 3' UTR F | CGCCCTAGGATTAATTTGAAGTTATAATTGTC | AvrII | Actin 3' UTR cloning into vector Pb238 |
| 4 | Actin 3' UTR R | GGGGTACCGACGTAAATATGCGAGCATG | KpnI | Actin 3' UTR cloning into vector Pb238 |
| 5 | hdhfr yfcu F | CGCGATATCCGAAATTGAAGGAAAAAACATC | EcoRV | Selection cassette cloning into Pb238 |
| 6 | hdhfr yfcu R | CGCCCTAGGTGCAGCCCAGCTTAATTCTTTTCG | AvrII | Selection cassette cloning into Pb238 |
| 7 | Actin 3' replacement F | CGGGATCCATTAATTTGAAGTTATAATTGTC | BamHI | Cloning of actin 3' UTR into proximal flanking region |
| 8 | Actin 3' replacement R | CGCGATATCGACGTAAATATGCGAGCATG | EcoRV | Cloning of actin 3' UTR into proximal flanking region |
| 9 | Cor delta C F | ATTTGCGGCCGCAAAATGGTGAATGATGTCCCTTGTATC | NotI | Cloning of truncated coronin (delta C) |
| 10 | Cor linker R | CACTAGTTCTAGATGCTGCTGCTGCATTAGT | XbaI | Cloning of truncated coronin (delta C) |
| 11 | ADF2 ORF F | GCTCTAGAGCAGCAGCAGCAATGGTTTCAGGAGTTAATGTATC | XbaI | ADF2 ORF cloning into vector Pb238 |
| 12 | ADF2 ORF R | GGACTAGTTTATGTGTTTAAAATAATAGCCTTAAGTTC | SpeI | ADF2 ORF cloning into vector Pb238 |
| 13 | UIS3 5' UTR F | GCCCGCGGACAATTTCATTTCGTTAGGGATCG | SacII | UIS3 5' UTR cloning into vector Pb238 |
| 14 | UIS3 5' UTR R | ATTTGCGGCCGCATACACTTTCATATATTTGTTATTTGTC | NotI | UIS3 5' UTR cloning into vector Pb238 |
| 15 | mCh ORF F | ATTTGCGGCCGCAAAATGGTGAGCAAGGGCGAGGAGGATAACATGG | NotI | mCherry ORF cloning into vector Pb238 |
| 16 | mCh ORF R | GCTCTAGATGCTGCTGCTGCCTTGTACAGCTCGTCCATGCCGCC | XbaI | mCherry ORF cloning into vector Pb238 |
| 17 | eGFP F | GACCACCCTGACCCACGGCGTGCAGTG | SacII | Cloning of rseGFP into Plasmogem formin1 tag |
| 18 | QCR1_134980 | GCCATCAGGGGGTGCAGAACC | ApaI | Cloning of rseGFP into Plasmogem formin1 tag |
| 19 | Pb actin ORF pEGFP F | TCGGGATCCATGGGTGATGAAGAAGTTCAGG | BamHI | Plasmodium actin ORF cloning into pEGFP-C1 |
| 20 | Pb actin ORF pEGFP R | GTTAtctagaTTAAAAGCATTTACGATGAAC | XbaI | Plasmodium actin ORF cloning into pEGFP-C1 |
| 21 | Oc beta actin ORF pEGFP F | TCGGGATCCATGGATGACGATATCGCCGCGCTCG | BamHI | Oryctolagus cuniculus beta actin ORF cloning into pEGFP-C1 |
| 22 | Oc beta actin ORF pEGFP R | GTTAtctagaCTAGAAGCACTTGCGGTGCACG | XbaI | Oryctolagus cuniculus beta actin ORF cloning into pEGFP-C1 |
|  |  |  |  |  |
| ***Genotyping primers- replacement (see fig S4)*** | | |  |  |
| **Primer no.** | **Primer name** | **Sequence** | **Used for** |  |
| Combination 1 | 5' UTR flanking F | GTGCTCATAAGATAATAACTTCA | Entire integration genotyping |  |
|  | 3' UTR flanking R | GTGATTGGGTTTTTCGTACTAG |  |  |
| Combination 2 | 5' UTR flanking F | GTGCTCATAAGATAATAACTTCA | 5' integration genotyping |  |
|  | Actin codon modified R | CTGAAAGGTGCTCAGGCTGC |  |  |
| Combination 3 | Actin codon modified F | CAATTCAGGCAGTTCTGAGCC | 3' integration genotyping |  |
|  | 3' UTR flanking R | GTGATTGGGTTTTTCGTACTAG |  |  |
|  |  |  |  |  |
| ***Genotyping primers- overexpression of actin binding proteins (see fig S8)*** | | |  |  |
| **Primer no.** | **Primer name** | **Sequence** | **Used for** |  |
| Combination 1 | UIS3 flanking F | GAAAGATATGTCATGGTTAAATTGTGC | 5' integration (profilin and ADF2) |  |
|  | mCherry R | TCACCTTCAGCTTGGCG |  |  |
| Combination 2 | Tg DHFR | CCCCGTTGTCTGAGAAGG | 3' integration (all) |  |
|  | UIS3 gene R | GTCCTGATGATAACAAAGCAATTGCAAC |  |  |
| Combination 3 | mCherry F | CCACAACGAGGACTACACCATCGTGGAA | mCh-ADF2 |  |
|  | ADF2 R | GGACTAGTTTATGTGTTTAAAATAATAGCCTTAAGTTC |  |  |
| Combination 4 | Profilin F | ATTTGCGGCCGCAAAATGGAAGAATATTCATGGG | profilin-mCh |  |
|  | mCherry R | TCACCTTCAGCTTGGCG |  |  |
| Combination 5 | UIS3 flanking F | GAAAGATATGTCATGGTTAAATTGTGC | 5' integration (coronin) |  |
|  | Coronin R | CACATTTATATATATTACATACTTGTTTTGGTAAAAATCC |  |  |
| Combination 6 | Coronin F | GGAATTCCATATGATGGTGAATGATGTCCC | coronin-mCh |  |
|  | mCherry R | TCACCTTCAGCTTGGCG |  |  |
|  |  |  |  |  |
| ***Genotyping primers- actin additional copy and formin 1 (see fig S9)*** | | |  |  |
| **Primer no.** | **Primer name** | **Sequence** | **Used for** |  |
| Combination 1 | 5' UTR flanking F | GAGCATACAAAAATACATGCACAC | Entire integration genotyping |  |
|  | 3' UTR flanking R | TGATTTACTTCCATCATTTTGCCC |  |  |
| Combination 2 | 5' UTR flanking F | GAGCATACAAAAATACATGCACAC | 5' integration genotyping |  |
|  | UIS3 promoter R | ATTTGCGGCCGCATGCATATACACTTTCATATATTTGTTATTTGTC |  |  |
| Combination 3 | hDHFR select cass F | CTAGCTAGCTTAATCATTCTTCTCATATACTTC | 3' integration genotyping |  |
|  | 3' UTR flanking R | TGATTTACTTCCATCATTTTGCCC |  |  |
| Combination 4 | mCherry F | CCACAACGAGGACTACACCATCGTGGAA | mCherry actin integration genotyping |  |
|  | Actin R | TTAGAAGCATTTTCTGTGG |  |  |
| Combination 5 | GT_124530 | TGGTAGGGCCTTGCATAGGTGA | Formin 1 rseGFP integration |  |
|  | GFP F | AGCTCGCCGACCACTACCAGCAGAAC |  |  |
| Combination 6 | QCR1_124530 | GGAGCAGTTCTCAGCAAAAAGCA | Integration flanking site inspection |  |
|  | QCR2_124530 | TGTAGATGGAGATTGATGAGGA |  |  |
